# Photophysical Investigation of Dyes and Dye-PMMA Systems: Insights into Absorption, Emission, and Charge Transfer Mechanisms

**DOI:** 10.1021/acs.jpca.4c05342

**Published:** 2025-01-28

**Authors:** Christina Kolokytha, Alexandra Sinani, Theodore Manouras, Evangelos Angelakos, Panagiotis Argitis, Nektarios N. Lathiotakis, Christos Riziotis, Demeter Tzeli

**Affiliations:** † Laboratory of Physical Chemistry, Department of Chemistry, National and Kapodistrian University of Athens, Zografou GR-15784, Greece; ‡ Theoretical and Physical Chemistry Institute, National Hellenic Research Foundation, 48 Vassileos Constantinou Ave., Athens 11635, Greece; § Department of Informatics and Computer Engineering, University of West Attica, Egaleo 12243, Greece; ∥ Institute of Electronic Structure and Laser, FORTH, 100 N. Plastira, Vassilika Vouton, Heraklion, Crete GR-70013, Greece; ⊥ Opticon ABEE, Tripolis 22100, Greece; # Institute of Nanoscience and Nanotechnology, NCSR Demokritos, Aghia Paraskevi 15310, Greece; ∇ Department of Materials Science and Technology, University of Crete, 710 03 Heraklion, Crete 700 13, Greece

## Abstract

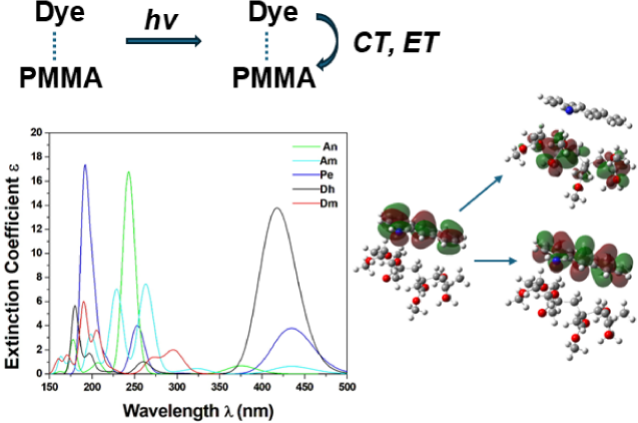

The photophysical properties of five dyes, i.e., perylene, anthracene, aminoanthracene, 1,6-diphenylhexatriene, and 7-diethylamino-4-methylcoumarin, in solvent and attached to the poly(methyl methacrylate) (PMMA) polymer, were studied via DFT and TD-DFT calculations. Their absorption and emission spectra were calculated, while for the PMMA-dye systems, their absorption spectra were measured experimentally, in good agreement with the calculated ones. In the PMMA-dye systems, charge transfer from the dye to PMMA was observed, and in the case of perylene, electron transfer from its ground state was also observed. It was found that the PMMA-dye systems can interact photochemically via laser illumination, and provided that the charge transfer will be enhanced by using the appropriate laser parameters, the systems may be candidates for the design of materials for specific nanopatterning needs.

## 1 Introduction

**Figure 1:**
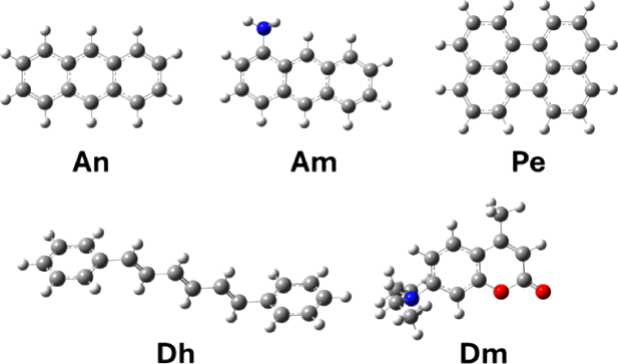
Molecular structures of the calculated dyes: anthracene (An), aminoanthracene (Am), perylene (Pe), 1,6-diphenylhexatriene (Dh), and 7-diethylamino-4-methylcoumarin (Dm). Gray spheres correspond to C atoms, white spheres to H atoms, red spheres to O atoms, and blue spheres to N atoms.

Dyes are colorants with various applications in material development,^1−3^ medicine,^4^ food,^5^ etc. During the last decades, many theoretical and experimental studies on dyes have been reported,^1−10^ while some of them present properties that are related to their photophysical and photochemical characteristics.^6−10^ Polymers such as PMMA are used as matrices in the gain medium of solid-state dye lasers, also known as solid-state dye-doped polymer lasers. These polymers have a high surface quality and are highly transparent, so the laser properties are dominated by the laser dye used to dope the polymer matrix.^11^ The combination of polymers and dyes is a research field of great potential for high-performance materials.^11−13^ This study aims to investigate the photochemical features of five different dyes, i.e., anthracene (An), aminoanthracene or anthramine (Am), perylene (Pe), 1,6-diphenylhexatriene (Dh), and 7-diethylamino-4-methylcoumarin or coumarin 1 (Dm), see Figure 1, and to examine the photochemical properties of mixtures of these dyes with the poly(methyl methacrylate) (PMMA) polymer to understand how their integration affects the composite PMMA spectra, with the aim of evaluating the potential of these dyes to sensitize PMMA after irradiation with the appropriate radiation.

The five studied dyes have many applications. Specifically, anthracene has luminescent properties^14−16^ and presents biological activity,^17^ while its main absorption UV–vis peak has been measured experimentally at 238 nm.^18^ Am also presents biological activity.^19^ Experimentally, its first absorption peak has been measured at 400 nm in glycerol/water/ethanol solution, 380 nm in glycerol/HCl,^20,21^ and 400 nm in ethanol, showing that the solvent affects the main peak^20,21^ and the main peak at 260–270 nm in ethanol.^20,21^ Experimental fluorescence spectroscopy studies report three main peaks near the region of 390, 406, and 409 nm.^20,21^ Pe can be used as an organic photoconductor and as a blue-emitting dopant material in OLEDs, while it displays blue fluorescence.^13^ Experimental studies for Pe have shown main absorption peaks at 444 and 253 nm and main emission peaks at 645 and 435 nm.^22^ Dh shows intense fluorescence when incorporated into the lipid bilayer.^23,24^ Dh presents unusual absorption and fluorescence properties in poly(vinyl alcohol) (PVA) film. Temperature affects both absorption and fluorescence properties of dyes in PVA film.^25^ Specifically, experimental studies on Dh in methylcyclohexane (dielectric constant ε = 2.02) solvent at 25 °C include an absorption spectrum with main peaks at 360 and 375 nm and fluorescence spectra with main peaks at ∼350, ∼360, and ∼380 nm.^26^ Finally, Dm is used as a fluorescent biosensor.^27^ Experimental studies on Dm present a broad UV–vis experimental absorption band between 300 and 450 nm^28,29^ with a peak at 370 nm,^29^ while the fluorescence spectrum has one main peak at ∼465 nm.^28^

**Figure 2:**
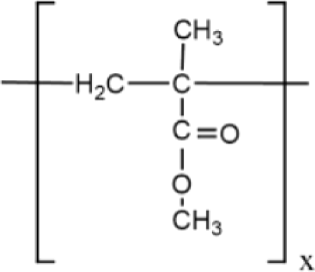
Molecular structure of methyl methacrylate, the monomer of PMMA.

PMMA is a synthetic photodegradable polymer^30^ derived from methyl methacrylate (see Figure 2). It is a transparent thermoplastic, mechanically strong and tough, lightweight, and versatile material that can be formed into many shapes, in pristine or composite synthesis, from nano/microwires^31^ to optical fibers^32^ or to widely known large-scale equipment used in automotive, furniture, electronics, and other industries.^33^ Furthermore, it presents many medical and dental applications where purity and stability are critical to performance.^33,34^ PMMA has attracted much attention in several studies for its use in laser-induced modification and ablation.^32,34−36^ In particular, PMMA doped with dye pigments is notable for its enhanced photosensitivity and ability to change optical properties.^37^ However, other approaches employing noble metal nanoparticles have been recently demonstrated toward the enhancement of material modification sensitivity.^38^ The addition of dopants expands the range of materials responsive to laser ablation by allowing strong absorption at specific design wavelengths. Upon laser excitation, these dopants undergo various processes, thus influencing material modification and the ablation mechanism. By changing the concentration and distribution of the dye molecules within the polymer matrix based on their absorption spectra and the laser wavelength used, the experimental conditions can be adjusted, making doped polymer composites a flexible system ideal for investigating the ablation mechanism in detail. The ablation process mechanism in such doped polymer systems has been attributed to a pure photothermal process in most of the cases, although sometimes the mechanism is not obvious or in detail investigated. In particular, a mechanism called multiphotonic cyclic absorption^39^ has been proposed, involving the absorption of multiple photons by the dye molecules, leading to a local increase in temperature, resulting in decomposition and finally, the ablation of the polymer.

The present computational study of the PMMA-dye systems can shed light on experimental research to distinguish if the system interacts photochemically with electromagnetic radiation or if a photothermal reaction takes place. During the direct laser exposure of materials, the photon absorption results in electron transfer (ET) or even in a charge transfer (CT) process from the dye to PMMA. Both CT and ET stand for charge transfer between the dye and PMMA. More specifically, ET designates the migration of a whole electron, while CT refers to the partial transfer of an electron. Photochemical reactions, facilitated by electron transfer, allow for the creation of precise and well-defined nanostructures due to the specific and localized nature of the interactions. By choosing a specific dye and the appropriate laser parameters, electron transfer can be enhanced, leading to the design of materials for specific nanopatterning needs with adequate control of spatial resolution due to the confinement and spatial restriction of the photochemical process. However, when the photon absorption is not accompanied by electron transfer between the dyes and PMMA, then, due to the sensitivity of the material and its interaction with electromagnetic radiation (illuminating light), a photothermal effect may occur, where the transfer and diffusion of the generated laser-induced heat will lead to wider ablation areas, depending also on the laser writing characteristics such as the laser pulse repetition rate^34^ and total delivered light fluence that govern the accumulated heat effect in a specific material. However, photothermally induced structures could provide adequate resolution for certain applications where high precision is not as critical, especially when femtosecond laser inscription is facilitated.^31^ Therefore, the study and identification of the electron transfer effects in PMMA-dye systems are critical for assessing the potential for laser-induced well-defined micro- and nanostructures.

## 2 Methods

### 2.1 Experimental Section

PMMA was dissolved in ethyl lactate at room temperature at a 3% w/w concentration, and then the dye was added to yield 5.0% w/w with respect to the PMMA weight solutions. The thin films were fabricated by spin coating the PMMA-dye solution at 2000 rpm for 120 s on a quartz substrate. After spin coating, the thin films were baked for 30 min at 100 °C in an oven to remove any residual solvent. UV–vis absorption spectra were recorded on a PerkinElmer Lambda 25 UV/vis spectrophotometer.

### 2.2 Computational

The molecular and electronic structures of the An, Am, Pe, Dh, and Dm dyes and the PMMA-dye systems, where the dyes are attached to PMMA, were studied using density functional theory (DFT). Five functionals were used, i.e., B3LYP,^40^ PBE1PBE,^41,42^ M06-2X,^43^ CAM-B3LYP,^44^ and ωΒ97XD^45^ functionals in conjunction with the 6-31G(d,p), 6-311+G(d,p),^46^ and def2-TZVP^47^ basis sets in CHCl_3_ solvent employing the polarizable continuum model (PCM).^48,49^ The CHCl_3_ solvent was used in the experimental study for the preparation of pigments, i.e., the polymer with each dye was dissolved in CHCl_3_ prior to the spin coating process. The dielectric constant of CHCl_3_, ε = 4.7113, and all calculated molecular systems are soluble in this solvent. Five hybrid functionals and three basis sets were used to check our methodology. Both 6-31G(d,p) and 6-311+G(d,p) basis sets result in the same geometries and similar absorption spectra. The def2-TZVP was used only in conjunction with the ωΒ97XD, so the ωΒ97XD/def2-TZVP data were used as benchmark DFT calculations for the interaction of the dye with the PMMA model system. Regarding the DFT functionals used, two of the functionals are long-range-corrected functionals, i.e., CAM-B3LYP, which uses the Coulomb-attenuating method, and ωΒ97XD, which includes empirical dispersion correction. Overall, the effect of the selected functionals on the calculation of the electronic structure, absorption spectra, and on the interaction energy between the dye and PMMA was evaluated.

At first, conformational analyses were carried out for the dyes, PMMA, and PMMA-dye systems. Geometry optimizations were performed on all species in their ground state to identify the lowest energy configuration for each. For the DFT calculations, the PMMA polymer was modeled with a molecule having four repeating subunits. Then, the absorption spectra of the studied structures were calculated via the time-dependent DFT (TD-DFT) methodology in the CHCl_3_ solvent. In all cases, the absorption spectra of the studied systems were calculated, including up to 50 singlet- and triplet-spin excited electronic states. The B3LYP emission spectra were calculated by optimizing each time the S_1_ state (first root). During optimization, only the singlet-spin transitions were considered, while 10 states were requested. The PCM model was used to include the solvent. All calculations were carried out employing the Gaussian16 code.^50^ The xyz geometries of the calculated dye molecules and PMMA-dye systems are given in the Supporting Information.

## 3 Results and Discussion

The photophysical properties of the five dyes and the PMMA-dye systems are studied here to investigate if the properties of the dyes are affected by the interaction with the PMMA polymer. The studied dyes were energetically optimized, both in the gas phase and in CHCl_3_ solvent. As expected, the dyes present a variety regarding their polarity. Specifically, three of them are symmetric (Pe, An, and Dh) having a dipole moment of 0 D, while the remaining two are polar. The dipole moment of Am is 1.672 (2.558) D in the gas phase (in CHCl_3_ solvent), while Dm has a significantly large dipole moment value of 9.160 (7.209) D, respectively. The differences in the values obtained in the gas phase and in solvent show that the solvent significantly affects the dipole moment of the dye. Finally, when the dyes interact with the PMMA model molecule, the PMMA-dye system has a nonzero dipole moment, i.e., all systems are polar; see Table 1. Regarding the PMMA, two model conformers have been calculated; one has the oxalic groups up and down with respect to the main carbon chain, and the other has the oxalic groups in the same direction. Both are polar, and their dipole moments are 3.557 and 1.371 D, respectively.

**Table 1:** Calculated Values of the Dipole Moment (D) for the Dyes and the PMMA-Dye Systems in the Gas Phase and in CHCl_3_ Solution Using the B3LYP/6-31G(d,p) Methodology

Dye	Dye in the gas phase	Dye in CHCl_3_ solvent	PMMA in CHCl_3_ solvent	Dye attached to PMMA
Am	1.672	2.558	5.714	2.211
An	0.000	0.000	2.125	0.036
Pe	0.000	0.000	4.159	0.003
Dh	0.002	0.003	4.390	0.103
Dm	9.160	7.209	7.133	8.910

### 3.1 Am Dye: Evaluation of the Computational Methodologies

**Figure 3:**
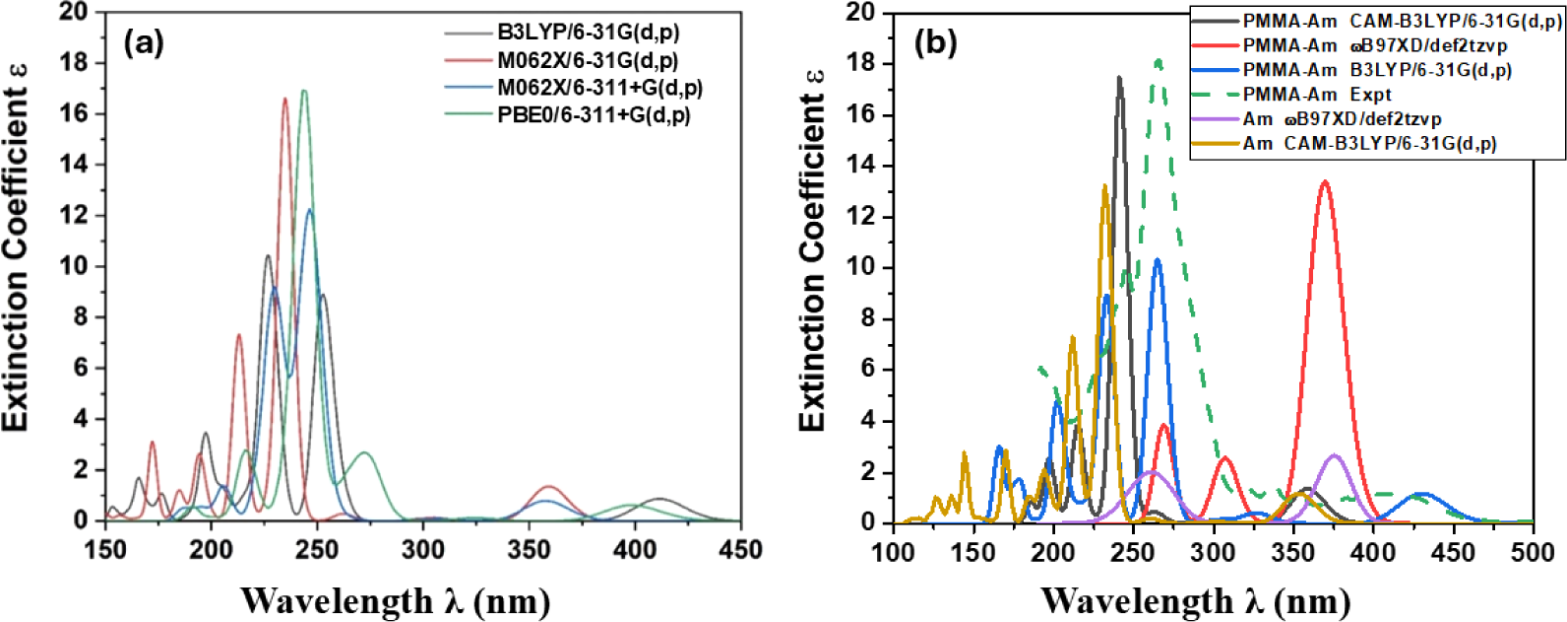
Absorption spectra of Am (a) in the gas phase using various methodologies and (b) in the CHCl_3_ solvent.

To find an appropriate methodology for the calculation of the absorption and emission spectra for one of the dyes, i.e., aminoanthracene (Am), its absorption spectrum was calculated using five different functionals and three basis sets, in the gas phase and in CHCl_3_ solvent, see Figure 3. Experimentally, the main excitation in the visible region corresponds to an S_0_ → S_1_ excitation, and it is located at 380–400 nm in different solvents,^20,21^ showing that there is a shift due to the solvent effect. The corresponding computational absorption peak of Am, depending on the methodologies used, ranges from 358 to 412 nm in the gas phase and 353 to 440 nm in CHCl_3_ solvent, see Table 2 and Figure 2. In the gas phase, B3LYP and PBE0 predict similar values, while the M06-2X functional results in significantly smaller λ than the B3LYP or PBE0 by about 45 nm. In CHCl_3_ solvent, the B3LYP predicts the S_0_ → S_1_ excitation at 440 nm, PBE0 at 430 nm, ωB97XD at 362 nm, and CAM-B3LYP at 353 nm, see Table 3. Thus, it seems that the functionals are grouped into two categories: the first group, consisting of the B3LYP and PBE0 functionals, predicts λ ≈ 435 nm, and the second one, consisting of ωB97XD and CAM-B3LYP, predicts λ ≈ 358 nm. Experimentally, we measured the absorption spectrum of the PMMA-Am system, see Table 4. The peak measured at 415 nm corresponds to an S_0_ → S_1_ excitation within the dye. The value of λ was calculated using B3LYP at 430 nm, while using CAM-B3LYP and ωB97XD at 359 and 369 nm, respectively. Furthermore, the Am dye presents an intense UV peak measured at 260–270 nm in various solvents.^20,21^ B3LYP predicts this peak at 252 (267) nm in the gas phase (in CHCl_3_ solvent), in excellent agreement with the experimental values. PBE0 predicts similar λ, while the M06-2X, CAM-B3LYP, and ωB97XD λ values are blue-shifted by about 30 nm with respect to the experimental values. Regarding the absorption spectrum of the PMMA-Am system, our experimental peak was measured at 271 nm. The B3LYP λ value is calculated at 267 nm, i.e., in excellent agreement with our experimental values. The ωB97XD predicts two absorption peaks at 307 and 269 nm, while the CAM-B3LYP predicts one absorption peak at 241 nm.

**Table 2:** Calculated Values of the Main Absorption Peaks, λ (nm), Energy Differences, Δ*Ε* (eV), Oscillator Strength, *f*, and Corresponding Main Excitations of the S_0_ → T_1_, S_0_ → S_1_, and S_0_ → S_*x*_ for Am in the Gas Phase, Using Various Methodologies^a^

	S_0_ → T_1_		S_0_ → S_1_				S_0_ → S_*x*_			
Meth^a^	λ	Δ*Ε*	λ	Δ*Ε*	*f*	Excit.	λ	Δ*Ε*	*f*	Excit.
1	710.6	1.745	411.5	3.013	0.064	H → L	252.4	4.913	0.631	H-2 → L
2	579.5	2.139	359.3	3.451	0.100	H → L	235.0	5.277	1.229	H-2 → L
3	557.8	2.223	357.7	3.466	0.070	H → L	229.8	5.395	0.714	H-2 → L
4	728.3	1.702	398.3	3.113	0.055	H → L	244.6	5.068	0.854	H → L+5
expt^b^			380–400				260–270			

a Methodology: 1: B3LYP/6-31G(d,p), 2: M062X/6-31G(d,p)//B3LYP/6-31G(d,p), 3: M062X/6–311+G(d,p) // B3LYP/6-31G(d,p), 4: PBE1PBE/6–311+G(d,p)//B3LYP/6-31G(d,p).

b Refs (20) and (21) in glycerol/water/ethanol, ethanol, and glycerol/HCl solution.

**Table 3:** Calculated Main Absorption Peaks, λ (nm), Energy Differences Δ*Ε* (eV), Oscillator Strengths, *f*, Coefficients, and Corresponding Main Excitations of the Dye Molecules in CHCl_3_, and the Available Experimental λ Values are Also Included

Dye	Meth^a^	λ	Δ*Ε*	*f*	State	Coef.	Excitations	Expt.
An	1	383.0	3.237	0.082	S_1_	0.9921	H → L	
		246.4	5.031	2.195	S_5_	0.7322	H → L+1	238^b^
		211.6	5.859	0.121	S_8_	0.9424	H-1 → L+1	
		180.2	6.880	0.364	S_16_	0.6948	H-2 → L+2	
Am	1	442.3	2.803	0.079	S_1_	0.9911	H → L	
		267.6	4.633	0.733	S_4_	0.6463	H-2 → L	270^c^
		233.4	5.312	0.766	S_6_	0.7434	H-1 → L+1	
		199.6	6.213	0.283	S_12_	0.6791	H-1 → L+2	
	2	353.2	3.510	0.104	S3	0.6992	H → L	
		232.2	5.339	1.174	S12	0.4658	H-2 → L	270^c^
		211.7	5.857	0.651	S16	0.5092	H-1 → L+1	
		169.7	7.304	0.137	S38	0.4222	H-1 → L+2	
	3	361.9	3.426	0.096	S1	0.6975	H → L	
		306.1	4.051	0.012	S2	0.5302	H → L+1	
		264.9	4.681	0.016	S3	0.6678	H-1 → L	270^c^
Pe	1	441.7	2.807	0.477	S_1_	0.9987	H → L	444^d^
		256.7	4.831	0.410	S_9_	0.7504	H → L+4	253^d^
		193.4	6.409	1.760	S_28_	0.6925	H-1 → L+3	
Dh	1	407.6	3.042	2.134	S_1_	1.001	H → L	375^e^
		264.0	4.696	0.103	S_6_	0.7549	H → L+2	
		182.2	6.804	0.590	S_25_	0.4203	H-3 → L+2	
Dm	1	296.2	4.187	0.260	S_2_	0.9369	H-1 → L	
		205.5	6.033	0.474	S_9_	0.6066	H-4 → L	
		189.5	6.544	0.504	S_13_	0.5144	H-2 → L+1	

a Methodology: 1: B3LYP/6-31G(d,p); 2: CAM-B3LYP/6-31G(d,p); 3: ωΒ97ΧD/def2-TZVP.

b Ref (18).

c Refs (20) and (21).

d Ref (22).

e Ref (26).

**Table 4:** Calculated Main Absorption Peaks, λ (nm), Energy Differences ΔΕ (eV), Oscillator Strengths, f, Coefficients and Corresponding Main Excitations of the PMMA-Dye Systems in CHCl_3_ Solution and Present Experimental λ_expt_ Values

Dye	Meth^a^	λ	Δ*Ε*	*f*	State	Coef	Main MO excit.	λ_expt_
An	1	383.8	3.230	0.103	S_1_	0.992	H → L	402
		247.2	5.017	1.855	S_7_	0.703	H → L+1	264
		212.1	5.845	0.144	S_20_	0.938	H-1 → L+1	
		183.0	6.776	0.006	S_42_	0.540	H-6 → L+5	
		181.0	6.852	0.444	S_44_	0.901	H-2 → L+4	
Am	1	430.1	2.883	0.104	S_1_	0.990	H → L	415
		266.5	4.653	0.609	S_7_	0.592	H → L+6	271
		233.9	5.301	0.694	S_10_	0.760	H-1 → L+1	
		232.2	5.339	0.024	S_11_	0.958	H-1 → L+2	
		200.4	6.186	0.217	S_32_	0.501	H-1 → L+6	
		166.0	7.467	0.003	S_85_	0.629	H-11 → L+2	
		165.6	7.486	0.099	S_86_	0.646	H-5 → L+6	
	2	358.6	3.457	0.121	S3	0.7001	H → L	415
		241.4	5.135	1.557	S12	0.5171	H-2 → L	271
		215.3	5.760	0.334	S20	0.1529	H-12 → L	
		196.5	6.310	0.206	S33	0.1087	H → L+4	
	3	369.4	3.356	0.119	S1	0.6983	H → L	415
		307.1	4.038	0.023	S2	0.5459	H → L+1	
		268.8	4.612	0.034	S3	0.6673	H-1 → L	271
Pe	1	443.4	2.796	0.517	S_1_	0.999	H → L	435
		257.3	4.820	0.477	S_11_	0.737	H → L+4	254
		255.1	4.860	0.002	S_12_	0.899	H → L+7	
		204.8	6.053	0.807	S_36_	0.753	H-4 → L+2	207
		196.2	6.320	0.002	S_44_	0.792	H-1 → L+6	
		193.9	6.396	1.501	S_47_	0.647	H-1 → L+3	
		192.2	6.450	0.012	S_49_	0.788	H-1 → L+7	
Dh	1	408.6	3.034	1.962	S_1_	1.001	H → L	377
		264.4	4.688	0.099	S_8_	0.593	Η-3 → L	
		182.6	6.790	0.581	S_64_	0.444	H-3 → L+3	
		177.4	6.989	0.004	S_77_	0.516	H-7 → L+6	
Dm	1	356.8	3.475	0.460	S_1_	0.989	H → L	363
		239.9	5.168	0.046	S_7_	0.569	H → L+5	
		227.8	5.443	0.250	S_12_	0.720	H-2 → L	243
		204.1	6.074	0.534	S_19_	0.914	H-1 → L+1	210

a Methodology: 1: B3LYP/6-31G(d,p); 2: CAM-B3LYP/6-31G(d,p); 3: ωΒ97ΧD/def2-TZVP.

Overall, B3LYP/6-31G(d,p) presents the best agreement regarding the calculation of the position of the main peaks, as observed in Figure 3b, where the blue line, which corresponds to the B3LYP/6-31G(d,p) absorption spectra, is in good agreement with the experimental one, i.e., the green dashed line. Note that both PBE0 and B3LYP present similar absorption spectra. Thus, for the calculation of the remaining dyes, one of them, i.e., the B3LYP functional, was chosen.

Regarding the geometry optimization of the ground state, all the above methodologies result in almost the same geometries for both dye molecules and the PMMA-dye system. The only difference is that the ωB97XD/def2-TZVP method predicts a small elongation of the van der Waals distance between PMMA and the dye compared to the other methodologies. This difference is expected since the ωB97XD functional includes dispersion corrections.

### 3.2 Absorption Spectra and Electronic Structures of Dyes

**Figure 4:**
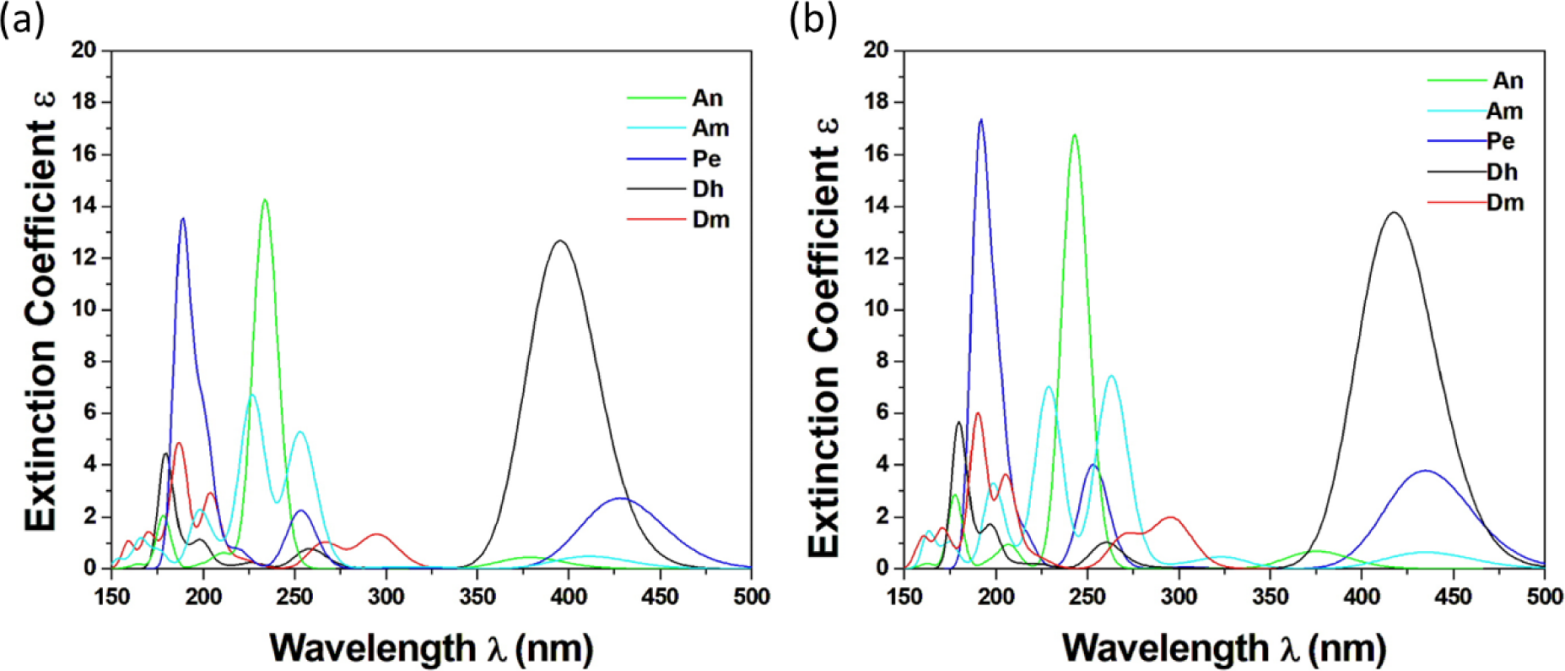
Absorption spectra of dyes (a) in the gas phase and (b) in CHCl_3_ solution using the B3LYP/6-31G(d,p) methodology.

The absorption spectra of the five studied dyes were obtained both in the gas phase and in CHCl_3_ solvent; see Figure 4. The general shapes of the absorption spectra are the same in both the gas phase and in CHCl_3_ solvent for all dyes. The absorption peaks of the dyes in the CHCl_3_ solvent are red-shifted with respect to the absorption peaks in the gas phase by ∼24 nm at most. For instance, in the gas phase, the absorption peak S_0_ → S_1_ of the Pe dye is at 428 nm (blue line in Figure 4) and that of the Dh dye is blue-shifted at 396 nm (black line), while in solvent, they are both red-shifted at 442 and 408 nm, respectively.

The calculated absorption spectra of the five dyes in CHCl_3_ solvent (see Figure 4) are in good agreement with available experimental absorption spectra. Specifically, in the case of An, the main peak in the experimental absorption spectra is 238 nm,^18^ which agrees with the present computationally obtained absorption peak at 246 nm. Similarly, there is excellent agreement in the case of Am, where the experimental main peak is in the area of 260–270 nm^20,21^ and the computational one at 268 nm. Moreover, the dye Pe has two peaks in the experimental absorption spectra at 253 and 444 nm,^22^ which are in concurrence with our computational main peaks at 257 and 442 nm. Recently, the vertical S_0_→S_1_ excitation (first peak) of the perylene clusters (perylene thin films) was calculated via the ω_T_B97X-D3/def2-SVP method where a tuned ωB97X-D3 functional was used.^51^ It was found that this value ranged from 407 nm (3.046 eV) to 449 nm (2.762 eV) depending on the size of the cluster, in good agreement with our values. In the case of Dh, the main peak from the experimental study is at 375 nm,^26^ which is red-shifted in the computational spectra.^52^ Finally, the last one, Dm, presents its first main peak in the UV area at 296 nm, in agreement with the recent computational value of 285 nm.^29^

**Figure 5:**
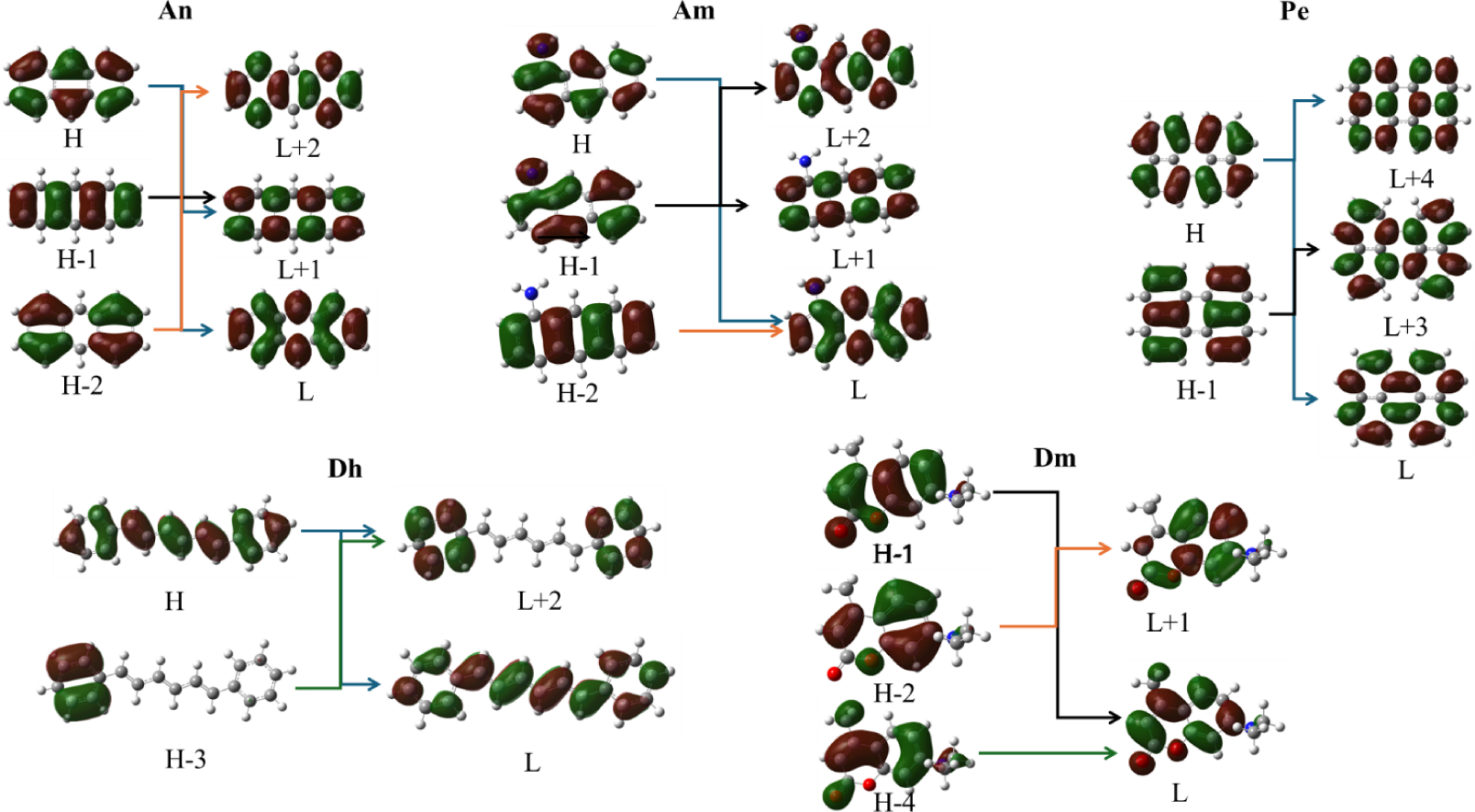
Frontier molecular orbitals of dyes involved in the main absorption excitations in CHCl_3_ solvent using the B3LYP/6-31G(d,p) methodology.

The frontier molecular orbitals (MOs) involved in the main absorption excitation peaks are depicted in Figure 5 and are analyzed below. For the An dye, the intense peaks, which correspond to H-2 → L+2 and H → L+1 excitations, are not electron transfer excitations. Similarly, the less intense peaks correspond to H → L and H-1 → L+1 MO excitations, which are also not electron transfer excitations. This behavior is probably due to the fact that this particular dye does not contain any strongly electronegative groups in its structure. In the case of the Am dye, both B3LYP and CAM-B3LYP functionals predict that the same MO is involved in the main absorption excitation peaks, even though the CAM-B3LYP excitation peaks are shifted to higher energies compared to the B3LYP, which presents the best agreement with the experimentally measured peaks among the methodologies used; see Table 3. Thus, the two main peaks of the Am dye correspond to H-2 → L and H-1 → L+1 excitations, and they present a CT character regarding the electron density in the amino group. On the other hand, there are two other absorption peaks, which are not intense; they correspond to H → L and H-1 → L+2 MO excitations, without any observed CT process.

Similarly, in the case of the Pe dye, no electron transfer is noted for the main excitations. The absence of a rich electronegative group, which tends to attract the electron cloud toward itself, leads to an equal distribution of the electron cloud throughout the molecular structure of Pe. In the case of the Dh dye, the main absorption peak at 407.6 nm is an S_0_ → S_1_ state excitation, which corresponds to an H → L MO electron excitation, where both H and L orbitals have electron density localized in the whole Dh dye. The two less intense absorption peaks correspond to H → L+2 and H-3 → L+2 MO excitations, and they can be characterized as electron transfer excitations within the dye Dh. Finally, the Dm dye presents three main excitations. The excitation H-1 → L is not an electron transfer one, while the H-4 → L and H-2 → L+1 excitations present very small CT character. Overall, intense main absorption peaks of the Am, Dh, and Dm present charge transfer character. This is in accordance with the fact that Am and Dm have strongly electronegative groups in their structure, while the Dh dye, even though it does not have electronegative groups, is less symmetric than An and Pe. Finally, it should be noted that weak absorption peaks for dyes present CT character.

### 3.3 Absorption Spectra of PMMA-Dye Systems

**Figure 6:**
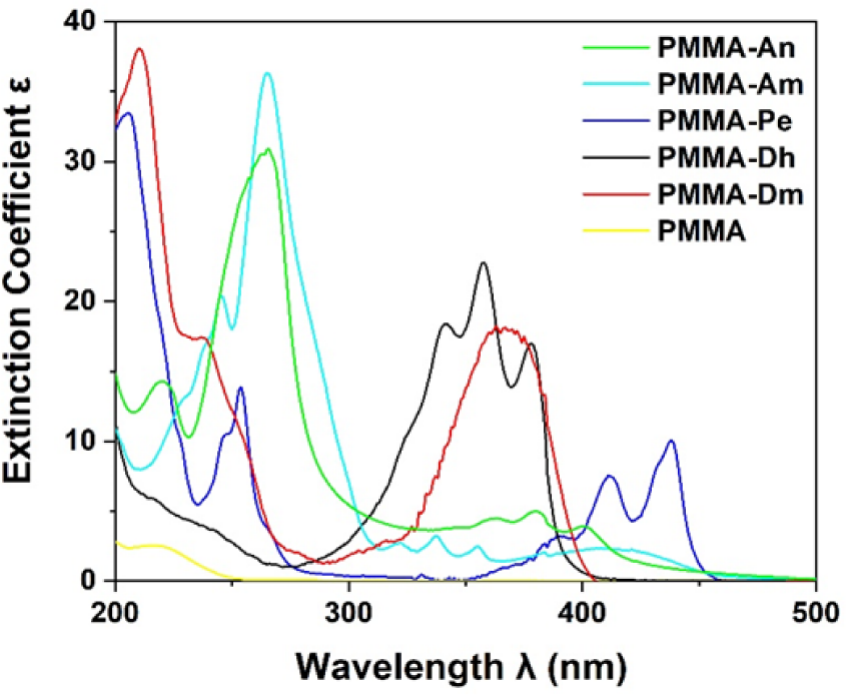
Experimental absorption spectra of PMMA and PMMA-dye systems.

Τhe UV–vis absorption spectra of the fabricated PMMA and PMMA-dye thin films have been measured, as shown in Figure 6. All dyes absorb in the UV–vis area, and broad bands are observed in all cases. PMMA-An presents a broad and rather weak band that ranges from 300 to 450 nm, with a weak peak at 402 nm, while An has an intense peak at 264 nm. The Am dye also presents a broad weak band that ranges from 320 to 450 nm, with its first weak peak at about 415 nm, while its intense peak is at 271 nm. The Pe presents two vis absorption peaks at 412 and 435 nm and two intense peaks at 254 and 207 nm. Dh and Dm have a strong broad band in the area of 300–400 nm, having three peaks at 340, 357, and 377 nm. Finally, the 7-diethylamino-4-methylcoumarin (Dm) presents a peak at about 363 nm, a shoulder at 243 nm, and an intense peak at 210 nm. It should be noted that PMMA does not absorb for λ > 250 nm, see Figure 6.

**Figure 7:**
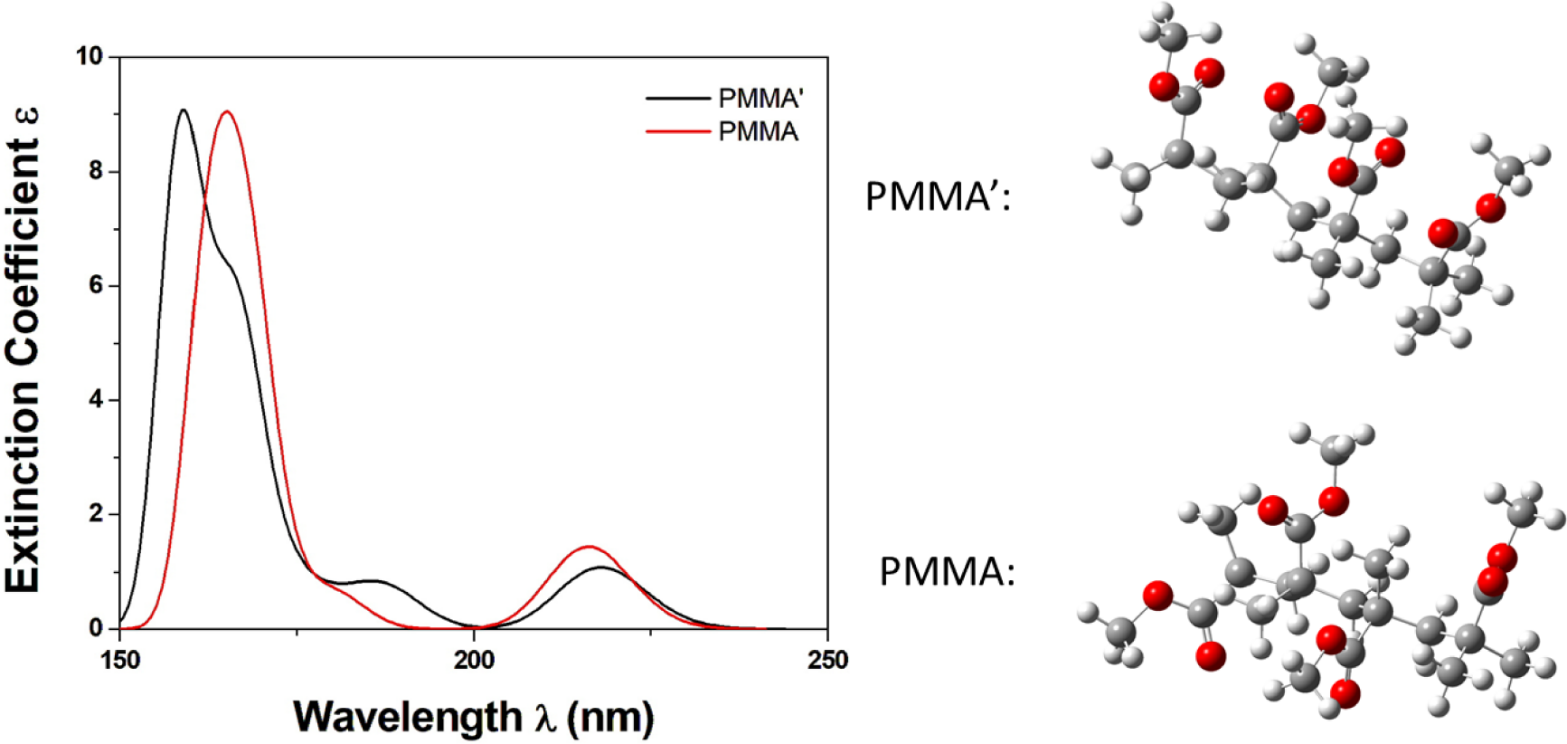
Absorption spectrum of the two lowest in energy conformers of the model PMMA structure in CHCl_3_ solution using the B3LYP/6-31G(d,p) methodology.

Computationally, the photochemical properties of the molecular structures of the PMMA-dye systems were studied. The PMMA polymer was modeled with a molecule having four repeating subunits. At first, conformation analysis was carried out to find the structures with the lowest energy. The two lowest-energy structures are depicted in Figure 7. They differ in the direction of the oxalic groups with respect to the main carbon chain. The conformer having the oxalic groups oriented up and down with respect to the main carbon chain is the lowest energy conformer. The PMMÁ conformer, with the oxalic groups on the same side, is higher in energy than the first conformer by 3.63 kcal/mol. Both have similar absorption spectra, while their S_0_ → S_1_ excitation differs by only ∼4 nm.

**Figure 8:**
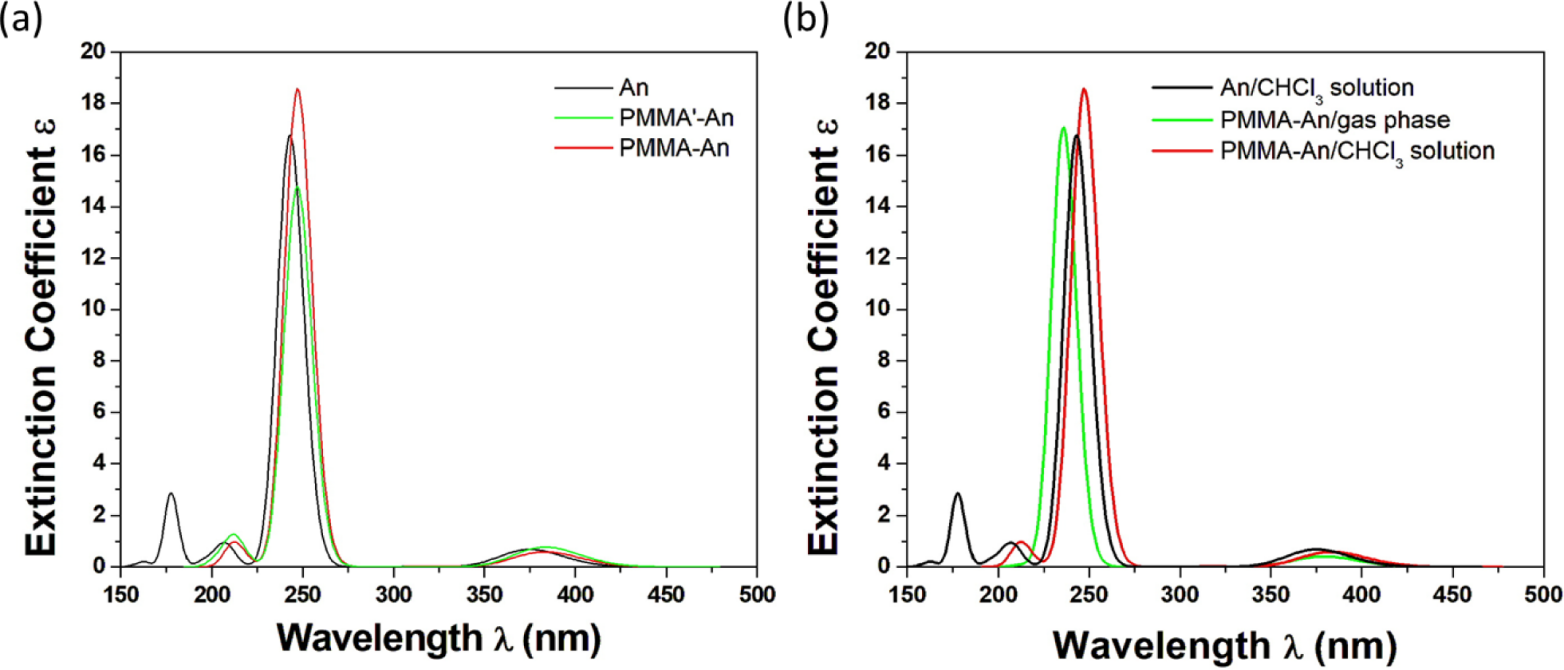
Absorption spectra of the (a) An, PMMA-An, and PMMA′-An structures in CHCl_3_ solution and (b) An in CHCl_3_ solution, and PMMA-An both in the gas phase and in CHCl_3_ solution using the B3LYP/6-31G(d,p) methodology.

In order to check the effect of the two PMMA isomers on the PMMA-dye molecular system for the An dye, both the PMMA-An and PMMÁ-An isomers were geometrically optimized, and their absorption spectra were calculated. The absorption spectra of PMMA-An and PMMÁ-An are the same; they present a main peak at 247 nm, which is located very close to the main peak of the free dye alone at 246 nm, see Figure 8. From the above, it can be concluded that the isomer of the polymer does not significantly affect the shape of the absorption spectra of the dye. As in the free dyes, the main absorption peak in the spectra of PMMA-An in CHCl_3_ solvent is red-shifted with respect to the corresponding peaks in the gas phase. Specifically, the main peak of the PMMA-An system in the gas phase is at 236.4 nm, while in the CHCl_3_ solvent, it is located at 247.2 nm.

**Figure 9:**
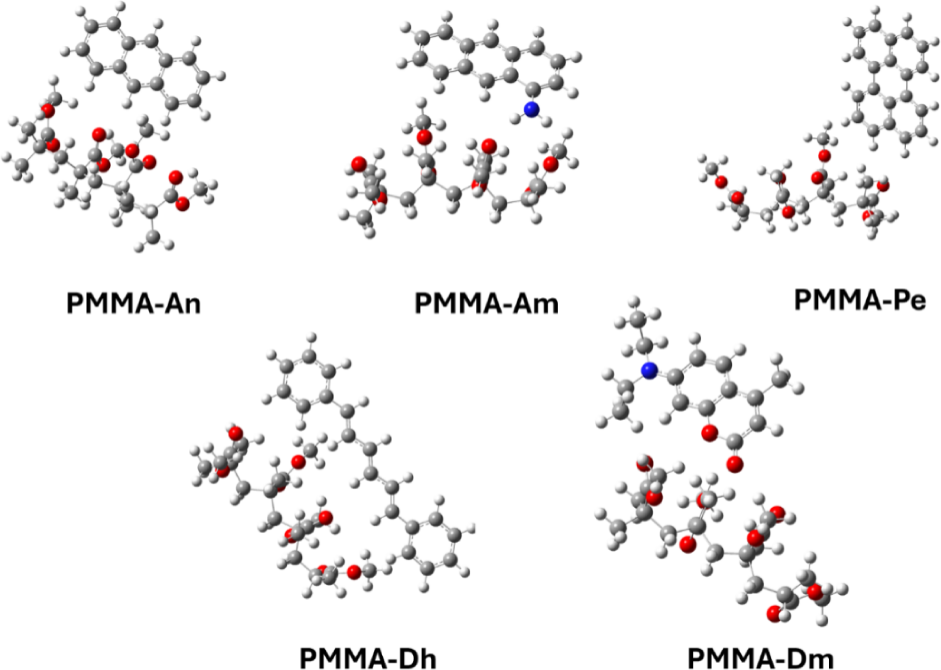
Calculated molecular structures of PMMA-dye systems. Gray spheres correspond to C atoms, white spheres correspond to H atoms, red spheres correspond to O atoms, and blue spheres correspond to N atoms.

Since it is found that the PMMA isomers result in almost the same absorption spectrum as the PMMA-dye, for the study of the PMMA-dye systems, the lowest energy PMMA conformer was used. It should be noted that van der Waals (vdW) interactions are formed between PMMA and the dyes in all PMMA-dye systems. The calculated PMMA-dye systems are listed in Figure 9. The corresponding vdW distances are about 2.4–2.7 Å, depending on the dye and methodology (B3LYP/6-31G(d,p), CAM-B3LYP/6-31G(d,p), and ωΒ97ΧD/def2-TZVP; see Tables S24 and S25). The ωΒ97ΧD/def2-TZVP is an appropriate methodology for the evaluation of van der Waals systems. The ωΒ97ΧD/def2-TZVP vdW distances were about 0.1 Å elongated with respect to the corresponding CAM-B3LYP/6-31G(d,p) and B3LYP/6-31G(d,p) vdW distances.

**Figure 10:**
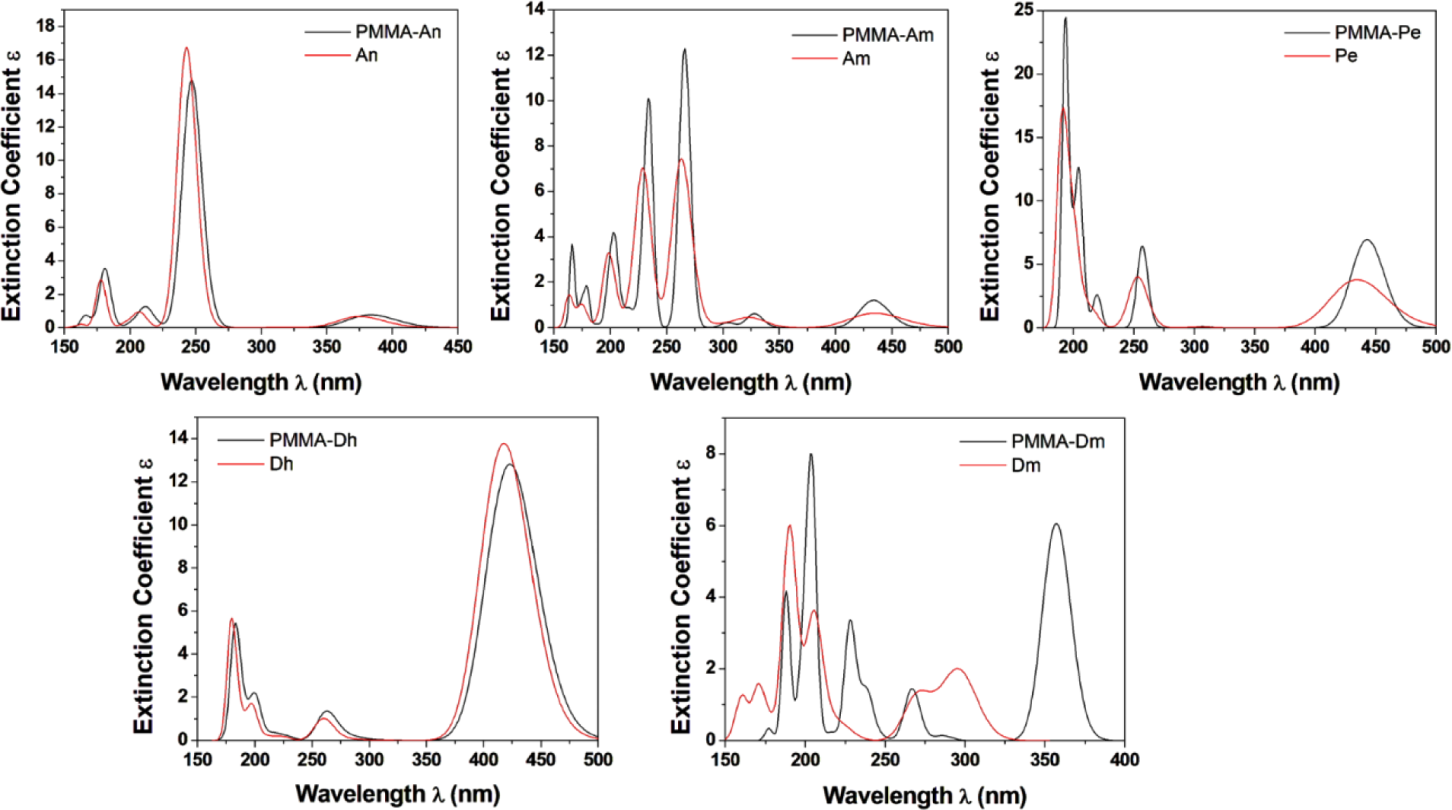
Absorption spectra for the dyes and PMMA-dye systems in CHCl_3_ solution using the B3LYP/6-31G (d, p) methodology.

The absorption spectra of all PMMA-dye systems and the corresponding dyes are depicted in Figure 10. It is found that the presence of the polymer does not affect the general shape of the absorption UV–vis spectra of most of the dyes. However, in the case of PMMA-Dm, the absorption spectrum shows an additional peak around 357 nm, which is not observed in the absorption spectra of the sole dye. The three main absorption peaks in the spectra of PMMA-Dm are at 357, 228, and 204 nm, while the main absorption peaks of Dm are at 296, 206, and 190 nm. Regarding the remaining dyes, the main absorption peaks of the free dyes compared to the PMMA-dye are shifted by up to 12 nm (Am vs PMMA-Am), as shown in Tables 3 and 4.

Comparing our experimental UV–vis absorption excitation peaks (Figure 6) with our calculated ones, see Table 4, there is very good agreement. The calculated absorption peaks are slightly shifted, i.e., the shifts range from 3 to 18 nm. Only for the PMMA-Dh system, there is a rather large red shift of the calculated first main peak with respect to the experimental ones of about 30 nm, which corresponds to 0.25 eV. Finally, the calculated absorption UV–vis spectra of the PMMA-Pe molecular system present the best agreement with the experimental ones, i.e., the observed shifts are less than 8 nm or less than 0.05 eV.

**Figure 11:**
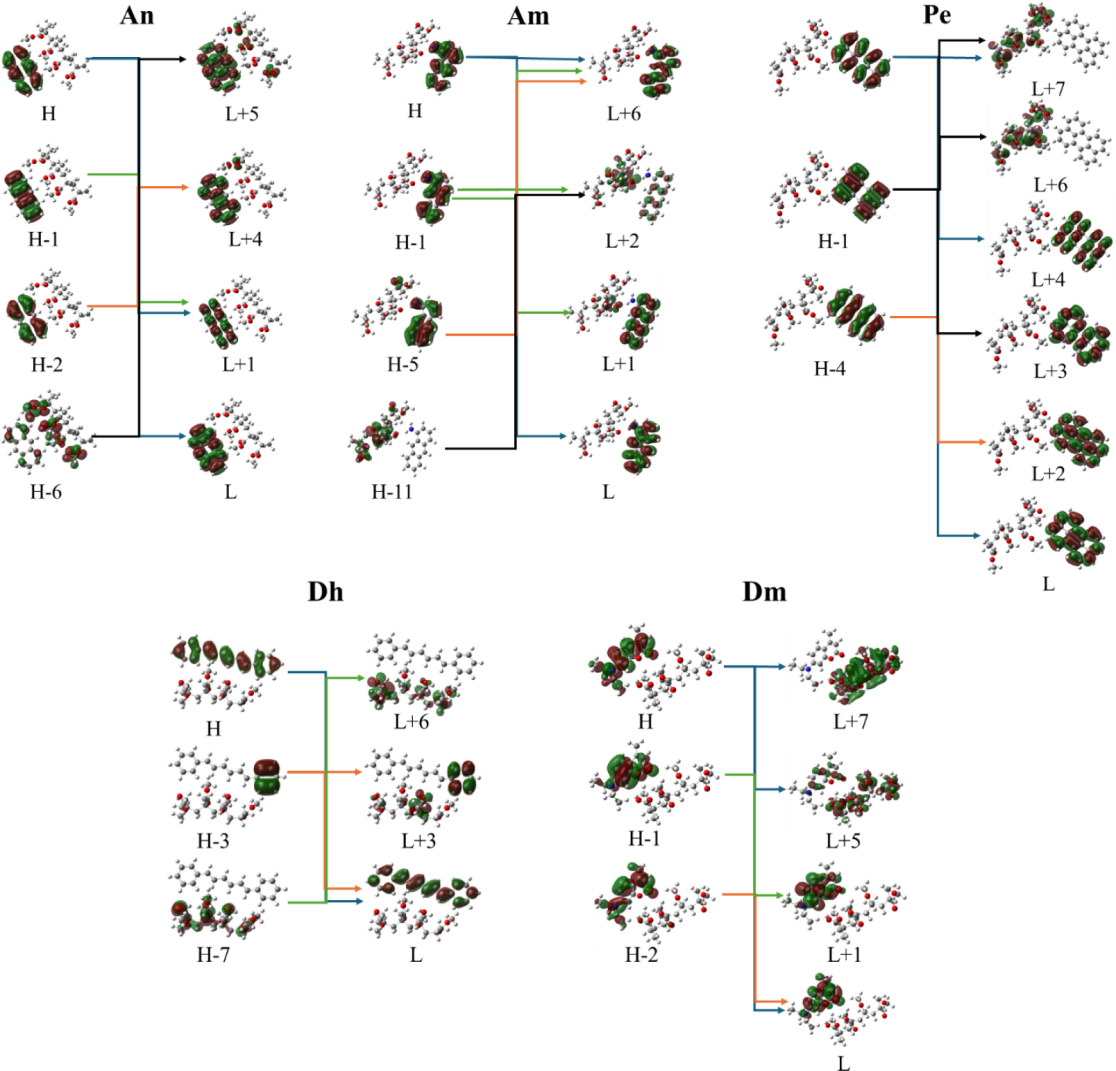
Frontier molecular orbitals for PMMA-dye systems at main excitations using the B3LYP/6-31G(d,p) methodology.

Even though the general shape of the absorption spectra of the free dyes and PMMA-dye systems is the same, absorption peaks corresponding to small charge transfers from the dye to the PMMA model system were found in the cases of An, Am, and Dh. In particular, in the PMMA-An system, the main peak at 247 nm and the peaks at 384 and 212 nm correspond to an electron excitation within the dye, while the peak at 181 nm corresponds to a very small charge transfer from the An dye to PMMA (H-2 → L+4 MO excitation), see Figure 11. This is likely due to the presence of polar groups in the polymer; i.e., the oxygen groups of PMMA attract the electron density.

**Figure 12:**
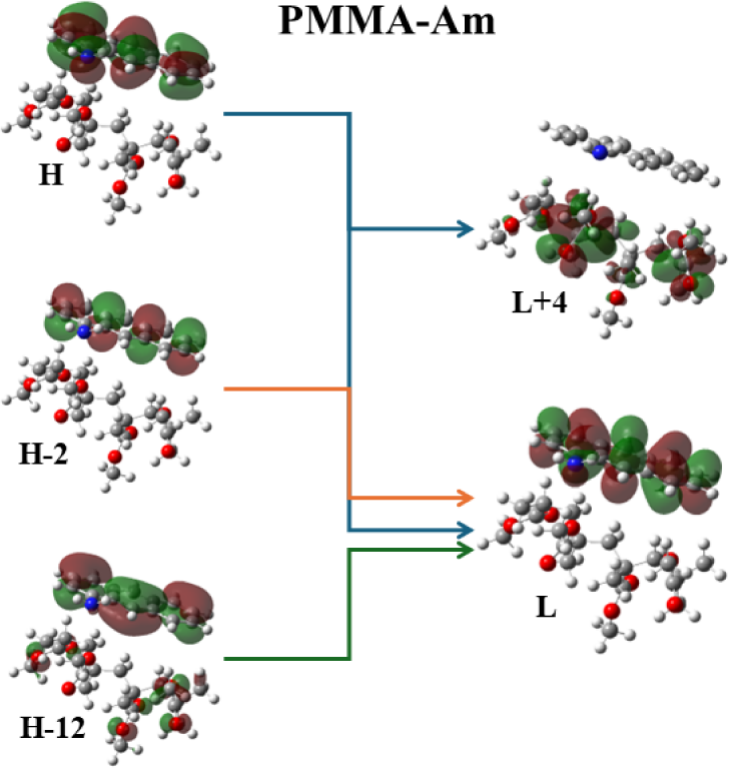
Frontier molecular orbitals of the PMMA-Am system at main excitations using the CΑΜ-B3LYP/6-31G(d,p) methodology.

Moreover, in the case of the PMMA-Am system, the main absorption peak at 234 nm corresponds to an H-1 → L+1 excitation, where a charge transfer from the nitrogen atom of Am to the PMMA is observed. On the contrary, the absorption peak at 232 nm, i.e., H-1 → L+2 MO, corresponds to a significant charge transfer from the dye to PMMA, while a small charge transfer is observed at 166 nm, i.e., H-5 → L+6 MO. However, for the absorption peaks at 430, 267, and 200 nm, a charge transfer process is not observed. Note that the B3LYP/6-31G(d,p) method presents the best agreement with the experimental absorption spectra. The CAM-B3LYP/6-31G(d,p) methodology, even though it results in blue shifts of the absorption peak compared to the experimental absorption peaks, up to 60 nm, presents a more intense CT process, as it is observed in the CAM-B3LYP MO (Figure 12) than the B3LYP/6-31G(d,p) or ωB97XD/def2-TZVP methodologies.

Furthermore, in the PMMA-Dh system, electron transfer is not observed from the dye to PMMA at the absorption peak of 409 nm (H → L MO excitation within the dye). The absorption peak at 264 nm corresponds to H-3 → L MO excitation, which corresponds to an electron transfer from one phenyl group to the whole dye. On the contrary, the absorption peak at 183 nm corresponds to an H-3 → L+3 MO excitation, where a charge transfer from the dye to the PMMA is observed. The PMMA-Dm system has three main absorption peaks at 357, 228, and 204 nm corresponding to electron excitations with dyes; however, the weak excitation at 240 nm corresponds to a CT process from the dye to PMMA.

Finally, the PMMA-Pe system has three main absorption peaks at 443, 257, and 204 nm, which do not correspond to any charge transfer excitation between the dye and PMMA. The weak excitations observed at 255, 196, and 192 nm indicate an electron transfer (ET) excitation from the dye to PMMA, suggesting that Pe and PMMA are capable of photochemical interaction.

**Figure 13:**
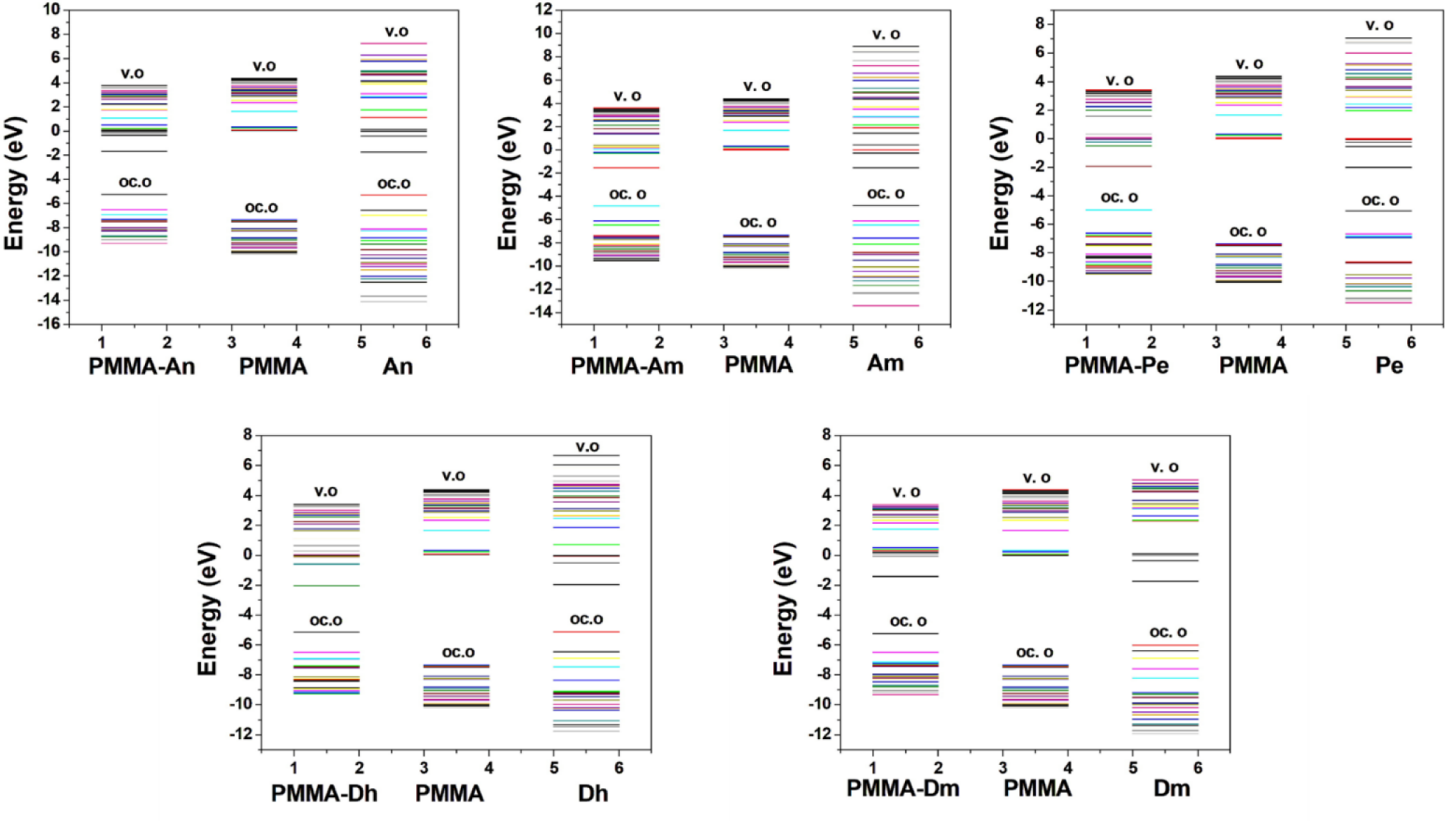
Energy diagram of molecular orbitals for dyes and PMMA-dye systems using the B3LYP/6-31G(d,p) methodology.

The molecular orbital energy diagrams of dyes, PMMA, and PMMA-dye are depicted in Figure 13. It is observed that the presence of the polymer does not significantly affect the molecular orbital energies of the dye, except for the Dm dye, where the presence of the polymer energetically stabilizes the H-2, H-1, and L molecular orbitals, while it destabilizes the H and the L+1 MOs. This is attributed to the fact that Dm is a very polar molecule (7.21 D), and when it is attached to PMMA, the PMMA-dye model also increases its polarity, affecting the relative energy of the MO.

To sum up, all five PMMA-dye systems present absorption UV–vis peaks that correspond to CT excitation from the dyes to PMMA. On the other hand, Pe is proven to be the optimal dye, as it exhibits ET excitations to PMMA at three different wavenumbers. Finally, it should be noted that the CT and ET excitations have a very small *f* value; however, under appropriately high laser illumination fluence, the population at the excited states will be increased.

### 3.4 Fluorescence Spectra of Dyes and PMMA-Dye Systems

**Figure 14:**
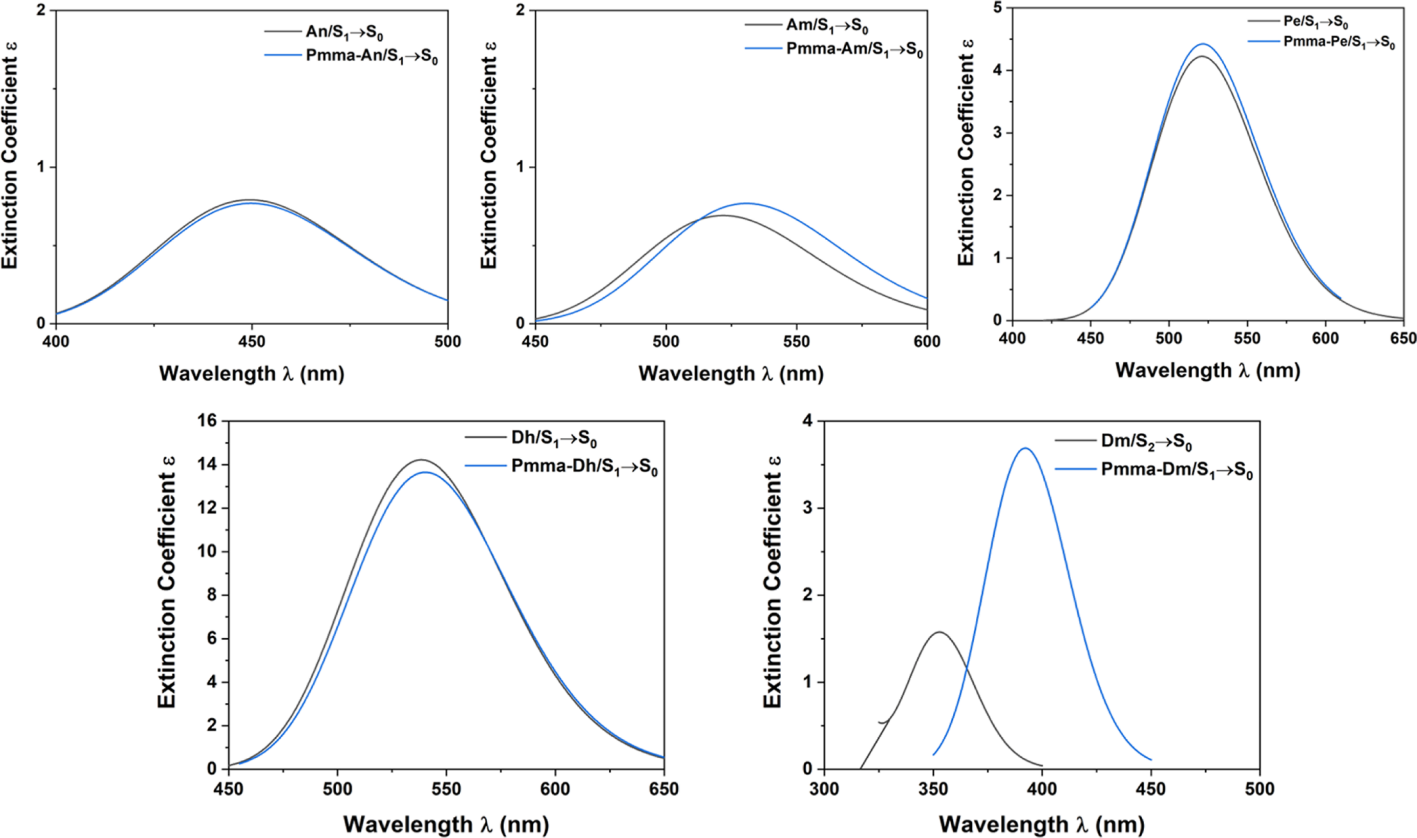
Emission spectra of dyes and PMMA-dye systems using the B3LYP/6-31G(d,p) methodology.

The main emission de-excitation peaks of the dyes and PMMA-dye molecular systems have been calculated; see Table 5, and the corresponding fluorescence spectra have been depicted in Figure 14. It seems that the presence of the polymer does not affect the general shape of the emission spectra of the An, Am, Pe, and Dh dyes. The largest shifts for the S_1_ → S_0_ de-excitation are around 8 nm, apart from Dm. Regarding Dm, for the free Dm, the S_1_ → S_0_ peak has an almost zero f (oscillator strength), and the fluorescence peak is attributed to an S_2_ → S_0_ de-excitation corresponding to H-1 → L MO, while in the PMMA-Dm, the fluorescence peak corresponds to the S_1_ → S_0_ de-excitation. As a result, a large red shift of 40 nm is observed due to PMMA; see Figure 12. The most intense fluorescence de-excitation is observed for the Dh and PMMA-Dh molecular systems, while the An, Am, PMMA-An, and PMMA-Am molecular systems present the least intense fluorescence peaks; see Table 5. Experimentally, the emission spectrum of anthracene in CHCl_3_ has been measured^51^ and presents strong peaks at 430 and 405 nm, and a broad low-intensity peak around 450 nm, in excellent agreement with our calculated S_1_ → S_0_ peak at 449.4 nm; see Table 3.

**Table 5:** Calculated Fluorescence S_1_ → S_0_ Peaks, Their^a^ λ (nm), Energy Differences Δ*Ε* (eV), Oscillator Strength, *f*, and MO Excitation of the Dyes and PMMA-Dye Systems in CHCl_3_ Solution by the B3LYP/6-31G(d,p) Methodology

	Dye			PMMA-Dye			
Dye	λ	Δ*E*	*f*	λ	Δ*E*	*f*	MO
An	449.4	2.759	0.106	449.7	2.757	0.103	H → L
Am	521.9	2.376	0.092	530.8	2.336	0.103	H → L
Pe	521.2	2.379	0.564	521.7	2.377	0.590	H → L
Dh	538.5	2.302	1.898	540.4	2.294	1.822	H → L
Dm^a^	352.9	3.513	0.210	392.2	3.161	0.492	H → L

a In the case of free Dm, S_2_ → S_0_ peaks corresponding to H-1 → L excitation.

## 4 Conclusions

In this work, five dyes, i.e., anthracene, aminoanthracene, perylene, 1,6-diphenylhexatriene, and 7-diethylamino-4-methylcoumarin, were studied in solvent and attached via van der Waals interactions to the PMMA polymer matrix, using DFT and TD-DFT calculations. Their UV–vis absorption and emission spectra were calculated, while experimentally, the absorption spectra of the PMMA-dye systems were measured.

There is good agreement between the theoretical and experimental results regarding the absorption spectra. Specifically, the B3LYP and PBE0 methods predict λ values in better agreement with the experimental ones than the M06-2X, CAM-B3LYP, and ωB97XD methods, where their λ values are blue-shifted by about 30–60 nm with respect to the experimental values. The B3LYP calculated absorption peaks are slightly shifted; i.e., the shifts range from 3 to 18 nm. Only for the PMMA-Dh system is there a rather large red shift of about 30 nm (0.25 eV). The calculated absorption UV–vis spectra of the PMMA-Pe system present the best agreement with the experimental ones; i.e., the observed shifts are less than 8 nm or less than 0.05 eV.

All five PMMA-dye systems present UV–vis absorption peaks that correspond to charge transfer excitation from the dyes to PMMA, at 181 nm (An), 232 nm (Am), 183 nm (Dh), and 240 nm (Dm), showing that all have the potential to interact photochemically and not only photothermally under the appropriate laser illumination conditions. However, Pe is found to be the best dye, as the excitations at 255, 196, and 192 nm indicate an electron transfer from Pe to PMMA, demonstrating that Pe can interact photochemically. The CT and ET excitations have a very small oscillator strength; however, with appropriately high laser light illumination fluence, the charge transfer excitations will be enhanced, as the population at the excited states will be increased.

Regarding fluorescence spectra, it was shown that the polymer does not affect the general shape of the emission spectra of the An, Am, Pe, and Dh dyes. It affects mainly the spectra of Dm, where a red shift of 40 nm is observed due to PMMA. This is attributed to the fact that the free Dm has an S_1_ → S_0_ peak with an almost zero oscillator strength (*f*), and the fluorescence peak is attributed to an S_2_ → S_0_ de-excitation corresponding to H-1 → L MO, while in PMMA-Dm, the fluorescence peak corresponds to the S_1_ → S_0_ de-excitation. Overall, the fluorescence peak of the PMMA-dye model system is observed in the area ranging from 540 to 392 nm.

The polarity of the dyes is affected by their attachment to PMMA. Specifically, the Dm dye, which is a highly polar compound, induces an increase of the polarity of PMMA. This interaction affects the relative energy of the dyes’ MO.

Overall, the present computational study of the PMMA-dye systems and their connection to conducted experimental measurements and other available experimental results provides an insightful investigation toward the identification of possible material modification mechanisms under laser light illumination. We base our reasoning on the assumption that when an electron transfer mechanism is observed, following a laser light illumination of a dye or a dye–polymer system, a photochemical process takes place in the material, possibly further to photothermal effects. In terms of laser material processing, the differentiation or selection of photothermal and photochemical processes is crucial as the intrinsically different mechanisms involved can lead to very different material processing qualities and characteristics. Indeed, photochemical processes lead to a confined material modification, which can be adjusted by proper selection of the material and the laser illumination conditions, while photothermal modification usually cannot be controlled accurately as the transfer of generated heat leads to accumulation effects and to wider modified or ablated areas. This effect is closely connected to the ability of laser micromachining systems to pattern specific materials accurately at a given resolution, which is a major challenge in photonics and micro/nano technology and has a great associated industrial impact. Therefore, by choosing a specific suitable dye and appropriate laser writing parameters, electron transfer can be enhanced, leading to a major photochemical modification component that could allow efficient laser-based nanopatterning.

## Supporting Information Available

Cartesian coordinates of the calculated systems, plots of their frontier molecular orbitals and calculated values of selected absorption peaks, energy differences, oscillator strengths, and corresponding main excitations at different levels of theory (PDF)
