# Supplementary material for: Photophysical Investigation of Dyes and Dye-PMMA Systems: Insights into Absorption, Emission, and Charge Transfer Mechanisms
Source: J Phys Chem A. 2025 Jan 28;129(5):1219–32. doi: 10.1021/acs.jpca.4c05342 (PMC11808785; doi:10.1021/acs.jpca.4c05342)
Supplement: Supplementary file 1 — jp4c05342_si_001.pdf [file jp4c05342_si_001.pdf]

Supporting Information

Photophysical Investigation of Dyes and Dye-PMMA Systems: Insights into Absorption, Emission, and Charge Transfer Mechanisms

Christina Kolokytha [a, b], Alexandra Sinani [b, c], Theodore Manouras [d,g], Evangelos Angelakos [e], Panagiotis Argitis [f], Nektarios N. Lathiotakis [b,*], Christos Riziotis [b], and Demeter Tzeli [a,b,*]

[a] *Laboratory of Physical Chemistry, Department of Chemistry, National and Kapodistrian University of Athens, Zografou GR-15784, Greece*

[b] *Theoretical and Physical Chemistry Institute, National Hellenic Research Foundation, 48 Vassileos Constantinou Ave., Athens, 11635, Greece*

[c] *Department of Informatics and Computer Engineering, University of West Attica, Egaleo, 12243, Greece*

[d] *Institute of Electronic Structure and Laser, FORTH, 100 N. Plastira, Vassilika Vouton, Heraklion, Crete GR-70013, Greece*

[e] *Opticon ABEE, Tripolis, 22100, Greece*

[f] *Institute of Nanoscience and Nanotechnology, NCSR Demokritos, Aghia Paraskevi, 15310, Greece*

[g] *Department of Materials Science and Technology, University of Crete, 710 03 Heraklion, Crete, Greece*

[*] Corresponding Authors: tzeli@chem.uoa.gr, lathiot@eie.gr

Table S1 Cartesian coordinates for An studied structures using B3LYP/6-31G (d, p) methodology.

	X	Y	Z
C	-4.86154	1.19089	-1.71878
C	-3.77179	0.60557	-1.12818
C	-3.84805	-0.72674	-0.61195
C	-5.09628	-1.44579	-0.72224
C	-6.20846	-0.79336	-1.34094
C	-6.09551	0.48319	-1.82498
C	-2.74648	-1.35163	-0.01174
C	-5.17513	-2.75198	-0.22252
C	-4.07237	-3.38396	0.36647
C	-2.82126	-2.66706	0.46662
C	-1.69349	-3.33004	1.0475
H	-0.74289	-2.80386	1.08524
C	-1.79967	-4.61317	1.51611
C	-3.03846	-5.31477	1.43334
C	-4.13763	-4.71971	0.87298
H	-1.82025	-0.79239	0.09163
H	-4.78914	2.19964	-2.11557
H	-2.82907	1.13884	-1.04282
H	-7.14776	-1.33439	-1.42279
H	-6.9478	0.96441	-2.29613
H	-6.11649	-3.29202	-0.29796
H	-0.93565	-5.10734	1.9509
H	-3.10294	-6.33039	1.81311
H	-5.08032	-5.2562	0.8012

Table S2 Cartesian coordinates for Am studied structures using B3LYP/6-31G (d, p) methodology.

	X	Y	Z
C	5.65899	-0.51148	0.64093
C	4.37248	-0.12207	0.97573
C	3.94929	1.23334	0.6798
C	4.8813	2.12361	0.01963
C	6.19167	1.66053	-0.30766
C	6.56033	0.37899	0.0064
C	2.67614	1.71143	1.01137
C	4.47091	3.42943	-0.28012
C	3.19501	3.90514	0.04238
C	2.27197	3.02065	0.71002
C	0.97141	3.51075	1.04331
H	0.27713	2.84181	1.54471
C	0.60114	4.79495	0.73562
C	1.51398	5.66892	0.07443

C	2.77024	5.2365	-0.26101
H	1.9661	1.0734	1.52832
H	5.98645	-1.51901	0.88661
H	6.88164	2.3396	-0.79929
H	7.55904	0.02531	-0.23518
H	5.17253	4.0959	-0.77682
H	-0.38992	5.15441	0.99658
H	1.2057	6.68366	-0.16036
H	3.46774	5.90165	-0.76367
N	3.51811	-0.98945	1.63486
H	3.76515	-1.9661	1.56041
H	2.51962	-0.84438	1.55345

Table S3 Cartesian coordinates for Am studied structures using M062X/6-311+G (d, p) methodology.

	X	Y	Z
6	4.097571	0.493084	-0.005487
6	2.983892	1.276739	-0.005537
6	1.677936	0.695953	-0.002056
6	1.555111	-0.732427	0.001493
6	2.749865	-1.519255	0.001407
6	3.976795	-0.928607	-0.001983
6	0.518155	1.473713	-0.002040
6	0.285410	-1.313449	0.005028
6	-0.874867	-0.537013	0.004921
6	-0.747246	0.891973	0.001367
6	-1.943975	1.676748	0.002711
1	-1.855529	2.757860	0.004130
6	-3.168135	1.091717	0.009064
6	-3.301782	-0.337206	0.003867
6	-2.178332	-1.117704	0.002181
1	0.604671	2.556416	-0.004307
1	5.082809	0.943676	-0.007989
1	3.068300	2.358365	-0.008017
1	2.656653	-2.600015	0.004329
1	4.873249	-1.537391	-0.001878
1	0.200017	-2.395905	0.008057
1	-4.067673	1.698178	0.024699
1	-2.266448	-2.199620	0.002726
7	-4.586678	-0.873722	0.063773
1	-5.310242	-0.307460	-0.353421
1	-4.661447	-1.843647	-0.204350

Table S4 Cartesian coordinates for Am studied structures using PBE1PBE/6-311+G (d, p) methodology.

	X	Y	Z
6	4.09649	0.48986	-0.00465
6	2.9798	1.27477	-0.00482
6	1.6787	0.69715	-0.00187
6	1.55536	-0.7339	0.00116
6	2.74597	-1.51683	0.00119
6	3.97602	-0.92542	-0.00158
6	0.51807	1.47295	-0.00187
6	0.28508	-1.31229	0.0042
6	-0.87664	-0.53763	0.00414
6	-0.74811	0.89359	0.00116
6	-1.94059	1.67361	0.00193
1	-1.85513	2.75632	0.00215
6	-3.16791	1.08824	0.00758
6	-3.30398	-0.33493	0.00369
6	-2.17476	-1.11487	0.00167
1	0.60477	2.55674	-0.00384
1	5.08247	0.94285	-0.00676
1	3.06677	2.3576	-0.00697
1	2.65532	-2.59922	0.00375
1	4.87275	-1.5368	-0.00133
1	0.19969	-2.39593	0.00697
1	-4.06553	1.70031	0.02102
1	-2.26225	-2.19828	0.00226
7	-4.5805	-0.87461	0.06303
1	-5.31595	-0.30456	-0.32401
1	-4.66054	-1.84257	-0.20595

Table S5 Cartesian coordinates for Am studied structures using M062X/6-31G (d, p) methodology.

	X	Y	Z
6	3.929411	-0.487886	0.021017
6	2.843084	-1.310848	0.050704
6	1.516987	-0.775071	0.027444
6	1.349987	0.647616	-0.022663
6	2.514652	1.476108	-0.058790
6	3.762972	0.928508	-0.036127
6	0.383235	-1.590750	0.035483
6	0.058064	1.182665	-0.039295
6	-1.072891	0.366848	-0.002053
6	-0.908076	-1.060586	0.012078
6	-2.064024	-1.902390	-0.016649
1	-1.926370	-2.979028	-0.016558
6	-3.307843	-1.349098	-0.053513
6	-3.486355	0.061926	-0.027367
6	-2.411604	0.911864	0.009614
1	0.508051	-2.671141	0.051461

1	4.930859	-0.905519	0.038629
1	2.964166	-2.389811	0.090791
1	2.381915	2.553692	-0.102843
1	4.639964	1.567018	-0.061681
1	-0.050516	2.260533	-0.119829
1	-4.187231	-1.984685	-0.081959
1	-4.492013	0.472961	-0.021042
7	-2.565340	2.300699	0.003985
1	-3.519952	2.593746	0.162959
1	-1.937085	2.783905	0.632884

Table S6 Cartesian coordinates for Pe studied structures using B3LYP/6-31G (d, p) methodology.

	X	Y	Z
C	4.43368	-0.9491	1.06315
C	5.49715	-1.96004	0.89871
C	7.70476	-2.60942	-0.01369
C	6.67256	-1.62551	0.14955
C	8.86967	-2.28661	-0.75491
C	9.01159	-1.03845	-1.31477
H	9.64308	-3.04078	-0.87088
C	8.00356	-0.07071	-1.15661
H	9.90298	-0.78976	-1.88317
H	8.15637	0.89945	-1.6143
C	5.77892	0.68337	-0.26884
C	4.60239	0.34735	0.47687
C	5.87936	1.96175	-0.80885
C	3.57069	1.33098	0.6399
C	4.86382	2.92167	-0.64671
H	6.75859	2.24524	-1.37546
C	3.72511	2.61838	0.06341
H	4.98853	3.9057	-1.08934
H	2.92678	3.34299	0.1919
C	6.83943	-0.32951	-0.43962
C	7.54777	-3.89289	0.56831
H	8.33698	-4.628	0.43726
C	6.41409	-4.19524	1.28583
H	6.29303	-5.17743	1.73323
C	5.3995	-3.23498	1.44769
H	4.52386	-3.51511	2.02121
C	3.26484	-1.21002	1.77305
H	3.11105	-2.18069	2.23005
C	2.25636	-0.24136	1.93074
H	1.36236	-0.49085	2.49456
C	2.40158	1.01023	1.37651
H	1.63637	1.77178	1.48972

Table S7 Cartesian coordinates for Dh studied structures using B3LYP/6-31G (d, p) methodology.

	X	Y	Z
C	7.58908	1.04914	0.61683
C	6.19596	1.0185	0.65318
C	5.43418	2.19574	0.50425
C	6.12955	3.40986	0.32402
C	7.5203	3.43901	0.28818
C	8.25956	2.26026	0.43362
H	8.15133	0.12714	0.73443
H	5.68049	0.07128	0.78874
H	5.5781	4.3389	0.21859
H	8.03294	4.38672	0.14996
H	9.3447	2.28825	0.40727
C	3.97649	2.09944	0.54593
H	3.58226	1.11234	0.78498
C	3.07572	3.08641	0.31287
H	3.41437	4.08972	0.05707
C	1.65268	2.87812	0.36946
H	1.31681	1.87522	0.63292
C	0.72337	3.83829	0.11516
H	1.05623	4.84479	-0.14086
C	-0.69612	3.60326	0.15728
H	-1.00374	2.58791	0.40141
C	-1.63603	4.55103	-0.08498
H	-1.29239	5.56101	-0.30916
C	-3.08812	4.3771	-0.06429
C	-3.71303	3.12134	0.0908
C	-3.91567	5.50862	-0.20618
C	-5.09968	3.00643	0.1157
H	-3.10839	2.22467	0.17712
C	-5.30462	5.39478	-0.1823
H	-3.45679	6.48619	-0.33139
C	-5.90372	4.14406	-0.01881
H	-5.54849	2.025	0.2394
H	-5.91932	6.28382	-0.29126
H	-6.98579	4.05381	0.00001

Table S8 Cartesian coordinates for Dm studied structures using B3LYP/6-31G (d, p) methodology.

	X	Y	Z
C	-5.43525	1.93245	-1.53097
C	-4.63487	3.01132	-1.21312
C	-3.61289	2.92473	-0.24648
C	-3.45277	1.6685	0.37463
C	-4.23131	0.56202	0.06743

C	-5.25668	0.66692	-0.89739
H	-6.21844	2.07047	-2.26459
H	-4.809	3.95515	-1.71978
C	-2.73335	4.00108	0.1407
H	-3.98741	-0.36655	0.56685
C	-1.62717	2.48296	1.73423
C	-1.78442	3.76818	1.09232
H	-1.0971	4.54078	1.41543
C	-2.86396	5.35561	-0.5026
H	-2.71736	5.29048	-1.58653
H	-3.86264	5.77527	-0.33703
H	-2.12787	6.05416	-0.10006
O	-0.81791	2.19604	2.60033
O	-2.48925	1.47569	1.32798
N	-6.05668	-0.40891	-1.21321
C	-7.02602	-0.35712	-2.30564
H	-6.61762	0.23964	-3.12699
H	-7.12713	-1.37386	-2.70024
C	-5.99147	-1.66973	-0.47038
H	-5.76504	-1.45814	0.5782
H	-6.99868	-2.1018	-0.48124
C	-4.987	-2.67892	-1.03667
H	-5.19076	-2.88347	-2.0932
H	-3.96223	-2.31624	-0.9373
H	-5.05796	-3.62412	-0.48835
C	-8.40482	0.17159	-1.89035
H	-8.83969	-0.44966	-1.10103
H	-8.33972	1.19535	-1.5113
H	-9.09145	0.16355	-2.74366

Table S9 Cartesian coordinates for PMMA-An studied structures using B3LYP/6-31G (d, p) methodology.

	X	Y	Z
6	-0.71253	-4.98697	1.64199
1	0.0313	-5.3444	2.36207
1	-1.41643	-5.80189	1.45134
1	-1.26612	-4.16451	2.09931
6	-0.00489	-4.54818	0.3424
6	0.98778	-3.40314	0.74554
1	1.77582	-3.88892	1.33454
1	0.44809	-2.74119	1.42798
6	1.70037	-2.46317	-0.28155
6	2.72675	-1.65188	0.60731
1	3.59708	-2.31095	0.70869
1	2.29664	-1.55089	1.60671
6	3.27291	-0.23077	0.22718

6	4.37594	0.06084	1.30596
1	5.0541	-0.80054	1.31749
1	3.87682	0.08264	2.27731
6	5.29061	1.32576	1.21767
1	5.6696	1.44725	2.23979
6	0.71697	-5.78012	-0.25073
1	1.15531	-5.57628	-1.22559
1	1.50807	-6.11109	0.43013
1	0.01222	-6.61007	-0.36928
6	2.45162	-3.23598	-1.38354
1	3.08257	-4.00462	-0.92515
1	1.75212	-3.71217	-2.06988
1	3.0961	-2.57907	-1.96668
6	3.83574	-0.1432	-1.19909
1	4.6763	-0.83459	-1.31524
1	3.07727	-0.38579	-1.94157
1	4.18213	0.86853	-1.41159
6	6.51282	1.17766	0.29846
1	7.08183	0.28696	0.58461
1	6.24518	1.08791	-0.75387
1	7.1748	2.0429	0.39776
6	-1.00774	-4.05502	-0.70972
6	0.64214	-1.51548	-0.86497
6	2.1436	0.80846	0.39416
6	4.4823	2.59954	0.99225
8	-0.73352	-3.89714	-1.88504
8	-0.29894	-1.07515	-0.22619
8	1.76042	1.56692	-0.47879
8	3.73894	3.08198	1.82338
8	-2.23845	-3.82323	-0.21597
8	0.87229	-1.19717	-2.1459
8	1.64504	0.79417	1.63843
8	4.6691	3.13096	-0.23252
6	-3.2079	-3.35079	-1.17075
1	-4.14037	-3.25268	-0.6175
1	-2.90343	-2.38067	-1.56779
1	-3.31019	-4.06213	-1.99323
6	-0.05601	-0.28196	-2.7561
1	0.23684	-0.23033	-3.80422
1	-1.076	-0.65809	-2.66172
1	0.02749	0.69882	-2.28543
6	0.68103	1.81327	1.96317
1	-0.2168	1.69924	1.35538
1	0.44653	1.66144	3.01629
1	1.12378	2.79844	1.80961
6	3.8532	4.27703	-0.54292
1	4.19804	4.6248	-1.51638
1	2.80466	3.97664	-0.5903

1	3.98037	5.05397	0.21369
6	-4.86154	-1.19089	1.71878
6	-3.77179	-0.60557	1.12818
6	-3.84805	0.72674	0.61195
6	-5.09628	1.44579	0.72224
6	-6.20846	0.79336	1.34094
6	-6.09551	-0.48319	1.82498
6	-2.74648	1.35163	0.01174
6	-5.17513	2.75198	0.22252
6	-4.07237	3.38396	-0.36647
6	-2.82126	2.66706	-0.46662
6	-1.69349	3.33004	-1.0475
1	-0.74289	2.80386	-1.08524
6	-1.79967	4.61317	-1.51611
6	-3.03846	5.31477	-1.43334
6	-4.13763	4.71971	-0.87298
1	-1.82025	0.79239	-0.09163
1	-4.78914	-2.19964	2.11557
1	-2.82907	-1.13884	1.04282
1	-7.14776	1.33439	1.42279
1	-6.9478	-0.96441	2.29613
1	-6.11649	3.29202	0.29796
1	-0.93565	5.10734	-1.9509
1	-3.10294	6.33039	-1.81311
1	-5.08032	5.2562	-0.8012

Table S10 Cartesian coordinates for PMMA-Am studied structures using B3LYP/6-31G (d, p) methodology.

	X	Y	Z
C	-6.99237	-0.69625	0.50138
H	-7.26613	0.16834	1.11377
H	-7.1795	-0.45241	-0.54725
H	-7.64435	-1.52932	0.783
C	-5.50116	-1.05705	0.74359
C	-4.66088	0.14826	0.2249
H	-4.85168	0.2348	-0.84833
H	-5.07653	1.04609	0.69372
C	-3.10516	0.21264	0.4199
C	-2.68464	1.50483	-0.36396
H	-3.11774	1.42335	-1.3646
H	-3.18464	2.34297	0.13065
C	-1.18596	1.9296	-0.54599
C	-1.27187	3.32536	-1.25682
H	-1.89466	3.96087	-0.62043
H	-1.81274	3.19064	-2.19812
C	0.00881	4.14393	-1.57871
H	-0.38626	5.03258	-2.09132
C	-5.30134	-1.34984	2.23913

H	-4.2958	-1.71531	2.45583
H	-5.47726	-0.4422	2.82278
H	-6.00357	-2.11776	2.57284
C	-0.43658	2.02633	0.79148
H	-0.94234	2.73081	1.4553
H	-0.37755	1.05565	1.28618
H	0.59153	2.36348	0.64819
C	-5.23538	-2.34069	-0.054
C	-0.45549	0.93434	-1.45618
C	-2.83642	0.46386	1.91299
C	0.6104	4.74898	-0.31218
C	-2.40953	-1.05008	-0.11178
H	-2.63731	-1.17572	-1.17401
H	-1.32651	-0.99878	0.00653
H	-2.74077	-1.94561	0.4164
C	1.01078	3.4889	-2.53777
H	1.5248	2.63055	-2.10379
H	0.48876	3.15435	-3.43923
H	1.77624	4.20748	-2.84066
O	-3.29754	1.40565	2.5301
O	-0.03357	5.38856	0.49588
O	-5.01985	-3.43566	0.42823
O	0.5786	0.34993	-1.17448
O	-5.32306	-2.13702	-1.38674
O	-2.03776	-0.46035	2.48151
O	-1.07646	0.76532	-2.63499
O	1.94166	4.5463	-0.18731
C	-1.75716	-0.27205	3.88372
H	-2.6823	-0.29971	4.46308
H	-1.10424	-1.09731	4.16338
H	-1.2585	0.68558	4.0464
C	2.55724	5.17759	0.95712
H	2.36904	6.2525	0.94332
H	2.16244	4.75457	1.88302
H	3.62352	4.97513	0.86635
C	-0.44996	-0.14397	-3.56422
H	0.55848	0.19572	-3.80733
H	-0.40084	-1.14793	-3.13787
H	-1.08253	-0.13492	-4.45035
C	-5.14782	-3.30427	-2.21205
H	-5.89944	-4.05944	-1.97248
H	-5.26705	-2.9589	-3.23821
H	-4.15322	-3.73116	-2.06501
C	5.42666	1.50164	0.78142
C	4.41285	0.77119	0.18622
C	4.5018	-0.67706	0.17521
C	5.63449	-1.31014	0.81806
C	6.64704	-0.50191	1.42047

C	6.53868	0.86425	1.38794
C	3.53502	-1.48074	-0.44086
C	5.71232	-2.70979	0.82564
C	4.73729	-3.5151	0.22432
C	3.61951	-2.88137	-0.43147
C	2.63075	-3.70553	-1.05315
H	1.78959	-3.22339	-1.54458
C	2.73481	-5.07234	-1.02755
C	3.83913	-5.69897	-0.3777
C	4.8092	-4.94386	0.22866
H	2.69398	-1.02884	-0.95688
H	5.36324	2.58672	0.7904
H	7.4943	-0.98755	1.8954
H	7.30764	1.48196	1.84401
H	6.56225	-3.18377	1.3115
H	1.97538	-5.68738	-1.50128
H	3.90587	-6.78299	-0.3666
H	5.65152	-5.42002	0.72386
N	3.35057	1.39669	-0.44832
H	3.24756	2.38452	-0.25621
H	2.45683	0.91886	-0.45721

Table S11 Cartesian coordinates for PMMA-Pe studied structures using B3LYP/6-31G (d, p) methodology.

	X	Y	Z
C	-1.22018	4.4543	-2.26529
H	-1.39529	4.1033	-3.28679
H	-2.06355	5.08001	-1.96332
H	-0.31666	5.07237	-2.27136
C	-1.04791	3.23492	-1.31818
C	-2.41211	2.48002	-1.31299
H	-3.1613	3.17155	-0.91752
H	-2.67093	2.29859	-2.36124
C	-2.60323	1.11936	-0.55474
C	-4.12861	0.8017	-0.736
H	-4.67771	1.69629	-0.4289
H	-4.29214	0.68253	-1.81117
C	-4.82171	-0.42205	-0.04295
C	-6.28025	-0.41014	-0.61851
H	-6.19224	-0.39503	-1.70874
H	-6.75001	0.53252	-0.32352
C	-7.27213	-1.55287	-0.27324
H	-8.21527	-1.22471	-0.73421
C	0.1252	2.38106	-1.82452
H	0.3739	1.57163	-1.13627
H	-0.12407	1.94875	-2.79729
H	1.02254	2.99398	-1.93963
C	-4.11162	-1.75108	-0.34163

H	-4.06572	-1.9198	-1.4197
H	-3.0973	-1.75917	0.06078
H	-4.63538	-2.59012	0.12147
C	-0.69384	3.80985	0.05918
C	-4.86617	-0.21819	1.47886
C	-1.80945	0.04699	-1.31901
C	-6.94436	-2.82007	-1.06161
C	-2.19699	1.22829	0.92302
H	-2.795	2.00191	1.41309
H	-2.34527	0.28859	1.45561
H	-1.14341	1.48988	1.03217
C	-7.5506	-1.77376	1.21884
H	-6.68616	-2.17784	1.74822
H	-7.82671	-0.82387	1.6866
H	-8.37951	-2.47172	1.35697
O	-1.98435	-0.20738	-2.49592
O	-6.70251	-2.83063	-2.25386
O	0.38476	3.70432	0.61505
O	-4.41458	-0.98634	2.30683
O	-1.71197	4.51713	0.59001
O	-0.89099	-0.58683	-0.56382
O	-5.4809	0.93506	1.81903
O	-6.98799	-3.9387	-0.31226
C	-0.10679	-1.59251	-1.23813
H	0.39426	-1.16382	-2.10793
H	0.62306	-1.93141	-0.50491
H	-0.74577	-2.41782	-1.55983
C	-6.72999	-5.17165	-1.01263
H	-5.72779	-5.16107	-1.44621
H	-6.81427	-5.95732	-0.26314
H	-7.46305	-5.3194	-1.80842
C	-5.56931	1.20762	3.231
H	-6.13392	0.42195	3.7374
H	-4.57166	1.27158	3.67059
H	-6.0857	2.16308	3.31317
C	-1.45047	5.14326	1.86107
H	-0.60978	5.83538	1.78088
H	-2.36376	5.67864	2.11706
H	-1.22329	4.38983	2.61849
C	4.62159	-1.02681	0.94751
C	5.73674	-1.98444	0.80388
C	8.02574	-2.49536	0.01036
C	6.94107	-1.56494	0.14859
C	9.22136	-2.0877	-0.63522
C	9.34266	-0.8083	-1.12668
H	10.03444	-2.80149	-0.73281
C	8.28237	0.10702	-0.99451
H	10.25718	-0.49418	-1.62075

H	8.42147	1.10364	-1.39617
C	5.96936	0.71955	-0.23097
C	4.76614	0.30028	0.4256
C	6.04256	2.02213	-0.71608
C	3.68191	1.23036	0.56331
C	4.97505	2.92903	-0.58012
H	6.93895	2.36747	-1.21745
C	3.81166	2.5464	0.04776
H	5.07982	3.93455	-0.9768
H	2.97604	3.23119	0.157
C	7.08611	-0.23666	-0.37132
C	7.88971	-3.81188	0.52098
H	8.71792	-4.50592	0.4093
C	6.72593	-4.19665	1.14602
H	6.61995	-5.20393	1.53752
C	5.66042	-3.28842	1.28486
H	4.76311	-3.63395	1.7842
C	3.42707	-1.36797	1.57621
H	3.29003	-2.3627	1.98368
C	2.36782	-0.45089	1.71072
H	1.45597	-0.76186	2.2118
C	2.48709	0.82782	1.2144
H	1.68143	1.5491	1.31044

Table S12 Cartesian coordinates for PMMA-Dh studied structures using B3LYP/6-31G (d, p) methodology.

	X	Y	Z
6	-5.5224	-3.32418	-0.99844
1	-5.28904	-3.70155	-1.99871
1	-5.39908	-4.13968	-0.28172
1	-6.57238	-3.01459	-0.99112
6	-4.59186	-2.12639	-0.66279
6	-3.1402	-2.69269	-0.60801
1	-3.1121	-3.43069	0.19852
1	-2.98316	-3.23915	-1.54347
6	-1.89624	-1.75605	-0.41412
6	-0.68981	-2.75327	-0.30374
1	-0.94829	-3.47221	0.47894
1	-0.66041	-3.30757	-1.24665
6	0.78039	-2.28106	-0.02645
6	1.62068	-3.60328	-0.10266
1	1.40179	-4.06115	-1.07183
1	1.24385	-4.28583	0.66449
6	3.16766	-3.57007	0.02954
1	3.44909	-4.63258	0.00331
6	-4.78933	-1.03732	-1.72914
1	-4.24191	-0.12326	-1.49195

1	-4.45186	-1.40394	-2.7021
1	-5.84606	-0.77012	-1.80767
6	1.27212	-1.24656	-1.04997
1	1.19865	-1.65178	-2.06151
1	0.69069	-0.32512	-0.9957
1	2.31038	-0.96773	-0.85993
6	-5.06162	-1.58687	0.69462
6	0.89476	-1.67658	1.38002
6	-1.7196	-0.94889	-1.71002
6	3.81643	-3.02693	-1.24234
6	-2.04037	-0.8736	0.83507
1	-2.15463	-1.5056	1.72021
1	-1.17218	-0.23046	0.98107
1	-2.91221	-0.22077	0.76951
6	3.71778	-2.98936	1.3379
1	3.54745	-1.9148	1.42139
1	3.23785	-3.48029	2.18973
1	4.79426	-3.16069	1.41242
8	-1.60434	-1.4596	-2.80792
8	3.5406	-3.42218	-2.35908
8	-5.60572	-0.51386	0.8782
8	1.30863	-0.56057	1.63925
8	-4.85673	-2.47414	1.69054
8	-1.70366	0.38661	-1.52234
8	0.48437	-2.53328	2.33662
8	4.75293	-2.08645	-1.00737
6	-1.54354	1.18641	-2.71403
1	-2.37346	1.01021	-3.40122
1	-1.53822	2.2192	-2.37061
1	-0.60375	0.93895	-3.21139
6	5.42297	-1.56428	-2.17358
1	4.69881	-1.12879	-2.86481
1	6.10378	-0.80099	-1.80045
1	5.97283	-2.35872	-2.6827
6	0.55939	-2.05003	3.69292
1	1.59044	-1.80245	3.95335
1	-0.06465	-1.16259	3.81664
1	0.19285	-2.86591	4.3142
6	-5.32161	-2.07544	2.99462
1	-6.39819	-1.89332	2.97785
1	-5.08507	-2.90716	3.65689
1	-4.81014	-1.16762	3.32197
6	7.58908	1.04914	0.61683
6	6.19596	1.0185	0.65318
6	5.43418	2.19574	0.50425
6	6.12955	3.40986	0.32402
6	7.5203	3.43901	0.28818
6	8.25956	2.26026	0.43362

1	8.15133	0.12714	0.73443
1	5.68049	0.07128	0.78874
1	5.5781	4.3389	0.21859
1	8.03294	4.38672	0.14996
1	9.3447	2.28825	0.40727
6	3.97649	2.09944	0.54593
1	3.58226	1.11234	0.78498
6	3.07572	3.08641	0.31287
1	3.41437	4.08972	0.05707
6	1.65268	2.87812	0.36946
1	1.31681	1.87522	0.63292
6	0.72337	3.83829	0.11516
1	1.05623	4.84478	-0.14086
6	-0.69612	3.60326	0.15728
1	-1.00374	2.58791	0.40141
6	-1.63603	4.55103	-0.08498
1	-1.29239	5.561	-0.30916
6	-3.08812	4.3771	-0.06429
6	-3.71303	3.12134	0.0908
6	-3.91567	5.50862	-0.20618
6	-5.09968	3.00643	0.1157
1	-3.10839	2.22467	0.17712
6	-5.30462	5.39478	-0.1823
1	-3.45679	6.48619	-0.33139
6	-5.90372	4.14406	-0.01881
1	-5.54849	2.025	0.2394
1	-5.91932	6.28382	-0.29126
1	-6.98579	4.05381	0.00001

Table S13 Cartesian coordinates for PMMA-Dm studied structures using B3LYP/6-31G (d, p) methodology.

	X	Y	Z
C	-0.29759	-4.84396	1.73187
H	0.34115	-5.56303	1.20972
H	0.09497	-4.70001	2.74144
H	-1.29983	-5.27756	1.81067
C	-0.33537	-3.50726	0.9417
C	1.11189	-2.93036	0.97098
H	1.35577	-2.73032	2.01768
H	1.77733	-3.73343	0.63771
C	1.50379	-1.64739	0.15668
C	2.98333	-1.36443	0.59711
H	2.9899	-1.3527	1.6906
H	3.56955	-2.2344	0.28564
C	3.77799	-0.09834	0.12348
C	5.22491	-0.32507	0.68222
H	5.54782	-1.31388	0.3435

H	5.16077	-0.3672	1.77311
C	6.37004	0.65876	0.32033
H	7.2173	0.30457	0.92566
C	-0.85541	-3.78909	-0.47643
H	-1.00303	-2.87178	-1.04968
H	-0.14567	-4.42497	-1.01273
H	-1.81795	-4.30476	-0.43379
C	3.78702	0.05796	-1.40454
H	4.20545	-0.836	-1.87283
H	2.78102	0.2234	-1.79288
H	4.38238	0.92166	-1.70865
C	-1.33738	-2.60399	1.67612
C	3.17543	1.17117	0.74826
C	1.52809	-2.03718	-1.33028
C	6.86477	0.42272	-1.10577
C	0.56537	-0.46872	0.45522
H	0.59246	-0.22562	1.5203
H	0.84681	0.42405	-0.10375
H	-0.46817	-0.69537	0.18854
C	6.13458	2.12923	0.68738
H	5.3529	2.58924	0.08051
H	5.84039	2.20379	1.73844
H	7.04808	2.7123	0.54922
O	2.19536	-2.95152	-1.77791
O	7.11161	-0.67782	-1.56217
O	-2.43072	-2.28031	1.24772
O	2.76032	2.13084	0.12545
O	-0.89226	-2.25491	2.8971
O	0.73469	-1.27063	-2.10553
O	3.16428	1.11339	2.09415
O	7.04144	1.56159	-1.80387
C	0.7245	-1.59784	-3.509
H	0.36251	-2.61706	-3.66005
H	0.04931	-0.87917	-3.97121
H	1.72854	-1.50755	-3.92868
C	7.54923	1.40267	-3.1432
H	6.8647	0.79771	-3.74154
H	7.62413	2.41042	-3.54943
H	8.52985	0.92221	-3.12564
C	2.61167	2.2586	2.78022
H	3.09542	3.17515	2.43681
H	1.53431	2.31987	2.6128
H	2.82024	2.08926	3.83624
C	-1.75835	-1.41726	3.69358
H	-1.35368	-1.46085	4.70443
H	-1.73107	-0.39247	3.31809
H	-2.7809	-1.79744	3.67173
C	-5.40322	2.03669	-1.48278

C	-4.57216	3.07707	-1.12207
C	-3.57113	2.92924	-0.13879
C	-3.46598	1.6519	0.45011
C	-4.27851	0.58283	0.10172
C	-5.2841	0.74816	-0.87849
H	-6.16568	2.22136	-2.2276
H	-4.70323	4.0392	-1.6062
C	-2.66616	3.96467	0.29314
H	-4.0822	-0.36726	0.58079
C	-1.63648	2.36808	1.85852
C	-1.74063	3.67311	1.25488
H	-1.03727	4.41624	1.61105
C	-2.74263	5.34088	-0.30994
H	-2.58132	5.30292	-1.39282
H	-3.73067	5.78547	-0.14745
H	-1.99193	6.00267	0.12575
O	-0.84734	2.02645	2.72853
O	-2.52342	1.40078	1.41411
N	-6.1155	-0.2858	-1.23465
C	-7.07155	-0.16898	-2.33791
H	-6.6327	0.43573	-3.13652
H	-7.20177	-1.17038	-2.76041
C	-6.1123	-1.56552	-0.52039
H	-5.9122	-1.38572	0.53967
H	-7.13063	-1.9647	-0.57177
C	-5.12494	-2.59217	-1.08562
H	-5.31365	-2.77011	-2.14936
H	-4.09139	-2.26055	-0.96378
H	-5.23507	-3.54528	-0.55793
C	-8.43484	0.3947	-1.92046
H	-8.89867	-0.23272	-1.15287
H	-8.34063	1.40655	-1.51605
H	-9.11001	0.43077	-2.78169

<u>Molecular structures for excited states optimization</u>

Table S14 Cartesian coordinates for An S_1□S_0 studied structures using B3LYP/6-31G (d, p) methodology.

	X	Y	Z
6	-4.38712	-1.0324	0.29472
6	-3.01749	-1.03222	0.29959
6	-2.28198	0.19406	0.29889
6	-3.01431	1.43981	0.29291
6	-4.44349	1.39353	0.28796
6	-5.10975	0.19687	0.28883
6	-0.88191	0.22703	0.30377
6	-2.30458	2.64712	0.29217

6	-0.90451	2.6801	0.29705
6	-0.17218	1.43434	0.30303
6	1.257	1.48062	0.30798
1	1.80595	0.54256	0.31247
6	1.92327	2.67728	0.3071
6	1.20063	3.90655	0.30121
6	-0.169	3.90637	0.29634
1	-0.33069	-0.71064	0.30827
1	-4.93257	-1.97152	0.29532
1	-2.46482	-1.96809	0.30409
1	-4.99243	2.33159	0.28347
1	-6.1956	0.17701	0.28502
1	-2.8558	3.58479	0.28767
1	3.00911	2.69714	0.31091
1	1.74608	4.84567	0.3006
1	-0.72166	4.84224	0.29185

Table S15 Cartesian coordinates for Am S_1□S_0 studied structures using B3LYP/6-31G (d, p) methodology.

	X	Y	Z
6	-7.88971	-1.06829	-0.04228
6	-6.50644	-1.12277	-0.0565
6	-5.74961	0.11448	-0.06997
6	-6.47499	1.36803	-0.0681
6	-7.90203	1.36099	-0.05316
6	-8.57778	0.16913	-0.04068
6	-4.35074	0.14798	-0.08465
6	-5.74953	2.56706	-0.08112
6	-4.35093	2.59782	-0.09582
6	-3.62891	1.34968	-0.09764
6	-2.20056	1.38286	-0.11263
1	-1.65833	0.44043	-0.11396
6	-1.52358	2.57409	-0.12511
6	-2.2371	3.8085	-0.12333
6	-3.60688	3.81888	-0.10913
1	-3.77285	-0.77241	-0.08641
1	-8.45671	-1.99564	-0.03218
1	-8.43235	2.30825	-0.05192
1	-9.66415	0.15935	-0.02927
1	-6.29733	3.50666	-0.07968
1	-0.43768	2.58486	-0.13644
1	-1.68475	4.74361	-0.13334
1	-4.15277	4.75877	-0.10775
7	-5.85425	-2.33411	-0.05783
1	-6.38247	-3.18852	-0.04864
1	-4.85474	-2.40603	-0.06849

Table S16 Cartesian coordinates for Pe S_1□S_0 studied structures using B3LYP/6-31G (d, p) methodology.

	X	Y	Z
6	1.38937	3.70102	5.8244
6	0.56095	3.63846	4.60381
6	0.0385	4.46944	2.33184
6	0.84375	4.53021	3.51823
6	0.31208	5.34854	1.25349
6	1.34259	6.25499	1.3417
1	-0.30539	5.29176	0.36138
6	2.13435	6.31813	2.50226
1	1.55343	6.92901	0.51674
1	2.93564	7.04699	2.52933
6	2.74167	5.5438	4.81215
6	2.45884	4.65209	5.89775
6	3.79453	6.44321	4.94782
6	3.2641	4.71286	7.08414
6	4.58097	6.4991	6.11236
1	4.03159	7.12941	4.14349
6	4.32413	5.65116	7.16421
1	5.39246	7.2183	6.17205
1	4.92577	5.68635	8.06817
6	1.91317	5.48133	3.5916
6	-1.02141	3.531	2.25169
1	-1.62302	3.49577	1.34771
6	-1.27817	2.68296	3.30349
1	-2.08954	1.96364	3.24371
6	-0.49178	2.73892	4.46806
1	-0.7288	2.05267	5.27237
6	1.16807	2.86435	6.91383
1	0.36672	2.13555	6.88679
6	1.95978	2.92755	8.07441
1	1.74882	2.25366	8.89945
6	2.99039	3.83389	8.16256
1	3.60784	3.89069	9.05468

Table S17 Cartesian coordinates for Dh S_1□S_0 studied structures using B3LYP/6-31G (d, p) methodology.

	X	Y	Z
6	-1.04427	10.31546	-2.57955
6	-0.55964	9.01319	-2.52846
6	0.21869	8.57137	-1.43854
6	0.48663	9.49438	-0.40861
1	-1.64156	10.63198	-3.42995
1	-0.78656	8.33216	-3.34268
1	1.08494	9.17538	0.44111

6	0.7546	7.21706	-1.32763
1	1.34368	7.02841	-0.43009
6	0.60274	6.19145	-2.20073
1	0.02175	6.33369	-3.1109
6	1.17203	4.88505	-2.00682
1	1.74954	4.73644	-1.095
6	1.01537	3.85388	-2.87783
1	0.39715	4.03417	-3.75682
6	1.56612	2.51707	-2.76896
1	1.16329	1.79795	-3.47946
6	2.52393	2.10394	-1.90475
1	2.96683	2.84005	-1.23533
6	3.072	0.75534	-1.77191
6	4.16279	0.54936	-0.90459
6	2.56662	-0.36354	-2.46585
6	4.73207	-0.71125	-0.74241
1	4.56617	1.39689	-0.35617
6	3.13605	-1.62179	-2.30496
1	1.71533	-0.2491	-3.12944
6	4.22259	-1.80466	-1.44372
1	5.57373	-0.8398	-0.06789
1	2.72797	-2.46823	-2.85009
1	4.6624	-2.78965	-1.31934
6	-0.76786	11.21687	-1.54674
1	-1.14827	12.23296	-1.59099
6	0.0011	10.79858	-0.46005
1	0.22335	11.48868	0.34882

Table S18 Cartesian coordinates for Dm S_1□S_0 studied structures using B3LYP/6-31G (d, p) methodology.

	X	Y	Z
6	-2.08072	-0.36599	0.22961
6	-0.72764	-0.11281	0.3944
6	-0.22841	1.20446	0.41526
6	-1.15503	2.25403	0.26251
6	-2.51943	2.00653	0.09609
6	-2.99374	0.69751	0.07814
1	-2.4389	-1.39044	0.21723
1	-0.03897	-0.9433	0.50957
6	1.17498	1.54962	0.58348
1	-3.20683	2.83601	-0.0189
6	0.56442	3.94396	0.42724
6	1.52423	2.86073	0.58536
1	2.55401	3.17686	0.70674
6	2.206	0.46694	0.75067
1	1.992	-0.14763	1.63239
1	2.2155	-0.20467	-0.11523

1	3.20501	0.89215	0.86455
8	0.81797	5.12562	0.42111
8	-0.76849	3.56235	0.26924
7	-4.40764	0.47944	-0.09558
6	-4.73692	-0.22263	-1.34816
1	-4.29186	-1.2347	-1.38779
1	-5.82364	-0.36426	-1.34969
6	-5.04695	-0.14394	1.07616
1	-6.10028	-0.29403	0.81349
1	-4.63443	-1.14776	1.29088
6	-4.9629	0.7247	2.32896
1	-3.92887	0.86755	2.65526
1	-5.39932	1.71031	2.14249
1	-5.51056	0.25149	3.15013
6	-4.33358	0.56497	-2.59239
1	-4.79482	1.55687	-2.58488
1	-3.2496	0.69517	-2.6567
1	-4.66106	0.03587	-3.49293

Table S19 Cartesian coordinates for PMMA-An S_1□S_0 studied structures using B3LYP/6-31G (d, p) methodology.

	X	Y	Z
6	-1.07208	4.80916	-2.15007
1	-1.42037	4.54652	-3.15378
1	-1.86732	5.35599	-1.63767
1	-0.2099	5.47548	-2.25697
6	-0.67998	3.51913	-1.3796
6	-1.98852	2.68838	-1.20857
1	-2.68118	3.29745	-0.62021
1	-2.42399	2.58482	-2.20788
6	-1.98221	1.25626	-0.56791
6	-3.4995	0.86128	-0.52109
1	-4.027	1.68426	-0.02963
1	-3.84153	0.83686	-1.56018
6	-4.00323	-0.46705	0.14157
6	-5.54169	-0.48469	-0.15878
1	-5.65918	-0.34687	-1.23762
1	-5.98816	0.3875	0.32801
6	-6.39776	-1.72275	0.21797
1	-7.42679	-1.40755	-0.01146
6	0.41711	2.78784	-2.16951
1	0.81856	1.93435	-1.6211
1	0.01647	2.43457	-3.12343
1	1.25356	3.46209	-2.37021
6	-3.30477	-1.71207	-0.42644
1	-3.44314	-1.76171	-1.50864
1	-2.23689	-1.7023	-0.20222

1	-3.7039	-2.62444	0.02209
6	-0.10136	3.97616	-0.03495
6	-3.77555	-0.42739	1.65943
6	-1.28738	0.31166	-1.56214
6	-6.17338	-2.86069	-0.77784
6	-1.3305	1.25619	0.82343
1	-1.87399	1.93997	1.48249
1	-1.33663	0.26384	1.27515
1	-0.2894	1.5795	0.7799
6	-6.36932	-2.14001	1.69398
1	-5.40031	-2.54208	1.99404
1	-6.59258	-1.27487	2.3263
1	-7.12064	-2.90782	1.89217
8	-1.65037	0.16296	-2.71127
8	-6.1313	-2.70901	-1.9812
8	1.05381	3.84998	0.31948
8	-3.14753	-1.24308	2.30231
8	-1.02596	4.6052	0.72757
8	-0.22291	-0.33773	-1.04141
8	-4.37123	0.64569	2.23601
8	-6.06809	-4.07032	-0.18516
6	0.46663	-1.22307	-1.94535
1	0.75461	-0.69045	-2.85375
1	1.34731	-1.56362	-1.40295
1	-0.17756	-2.06355	-2.2148
6	-5.89892	-5.18066	-1.08344
1	-4.98598	-5.06174	-1.67155
1	-5.83503	-6.06439	-0.44913
1	-6.74904	-5.25576	-1.76558
6	-4.20457	0.75023	3.65976
1	-4.62174	-0.12617	4.1613
1	-3.14599	0.83125	3.91798
1	-4.74146	1.65211	3.95347
6	-0.54946	5.10864	1.98698
1	0.25879	5.82732	1.83328
1	-1.40777	5.59082	2.45442
1	-0.17943	4.29231	2.61204
6	10.07911	-1.94395	-0.12861
6	9.29706	-0.8241	-0.23059
6	7.89246	-0.88059	0.03152
6	7.30995	-2.14836	0.40811
6	8.16292	-3.29213	0.50274
6	9.50428	-3.19489	0.24312
6	7.06691	0.24672	-0.06392
6	5.93522	-2.21565	0.66711
6	5.10987	-1.08839	0.57102
6	5.69219	0.17982	0.19508
6	4.83961	1.32446	0.1024

1	5.28179	2.27709	-0.1781
6	3.49724	1.23227	0.3606
6	2.92392	-0.02178	0.73024
6	3.70477	-1.14362	0.83231
1	7.50554	1.20113	-0.34672
1	11.14461	-1.88558	-0.33074
1	9.73318	0.13045	-0.51365
1	7.72276	-4.24464	0.78648
1	10.13973	-4.07237	0.3192
1	5.49669	-3.16979	0.95126
1	2.8612	2.10974	0.28954
1	1.85757	-0.08047	0.92607
1	3.26861	-2.09685	1.12097

Table S20 Cartesian coordinates for PMMA-Am S_1□S_0 studied structures using B3LYP/6-31G (d, p) methodology.

	X	Y	Z
6	6.48498	0.14515	1.56001
1	6.32872	-0.12316	2.60949
1	6.85196	-0.73542	1.02678
1	7.25938	0.91812	1.52169
6	5.15582	0.66636	0.94937
6	4.15561	-0.52788	0.97136
1	4.5744	-1.30956	0.33093
1	4.15421	-0.91889	1.99406
6	2.65347	-0.34675	0.55797
6	2.07773	-1.80501	0.59712
1	2.74009	-2.42365	-0.01538
1	2.18326	-2.14923	1.63053
6	0.60733	-2.1445	0.17054
6	0.46774	-3.67629	0.48294
1	0.71283	-3.80167	1.54192
1	1.23545	-4.20677	-0.08898
6	-0.87527	-4.42477	0.25505
1	-0.63829	-5.46027	0.53695
6	4.68218	1.88281	1.7608
1	3.80632	2.35683	1.31416
1	4.43335	1.57671	2.78059
1	5.46927	2.63956	1.8036
6	-0.43531	-1.31697	0.93761
1	-0.32211	-1.47254	2.0127
1	-0.33426	-0.25233	0.72233
1	-1.44975	-1.59528	0.64781
6	5.4886	1.12352	-0.4768
6	0.41644	-1.90871	-1.33162
6	1.96382	0.45642	1.67421
6	-1.90661	-4.02344	1.30866

6	2.51499	0.30408	-0.8271
1	3.02351	-0.31148	-1.57505
1	1.47018	0.41546	-1.11991
1	2.95502	1.30233	-0.84906
6	-1.39304	-4.45567	-1.18789
1	-1.70533	-3.47194	-1.53999
1	-0.60834	-4.82447	-1.85579
1	-2.25267	-5.12496	-1.27483
8	1.97393	0.12253	2.84207
8	-1.69506	-4.07683	2.50161
8	5.4919	2.27209	-0.86629
8	-0.44808	-1.21492	-1.83985
8	5.84546	0.08429	-1.27251
8	1.34383	1.56991	1.23339
8	1.32065	-2.57543	-2.07653
8	-3.08568	-3.61807	0.78445
6	0.68114	2.35649	2.24407
1	1.40431	2.7054	2.98475
1	0.23125	3.19178	1.71038
1	-0.08307	1.76012	2.74702
6	-4.08278	-3.22659	1.75104
1	-4.42351	-4.09781	2.31577
1	-3.67076	-2.49202	2.44502
1	-4.89824	-2.79101	1.17577
6	1.20328	-2.40938	-3.50179
1	0.22722	-2.75542	-3.8488
1	1.32419	-1.35883	-3.7749
1	2.00116	-3.01343	-3.9325
6	6.23512	0.44075	-2.60873
1	7.08745	1.12406	-2.59256
1	6.50423	-0.49576	-3.09719
1	5.4087	0.92499	-3.13496
6	-5.65899	-0.51148	-0.64093
6	-4.37248	-0.12207	-0.97573
6	-3.94929	1.23334	-0.6798
6	-4.8813	2.12361	-0.01963
6	-6.19167	1.66053	0.30766
6	-6.56033	0.37899	-0.0064
6	-2.67614	1.71143	-1.01137
6	-4.47091	3.42943	0.28012
6	-3.19501	3.90514	-0.04238
6	-2.27197	3.02065	-0.71002
6	-0.97141	3.51075	-1.04331
1	-0.27713	2.84181	-1.54471
6	-0.60114	4.79495	-0.73562
6	-1.51398	5.66892	-0.07443
6	-2.77024	5.2365	0.26101
1	-1.9661	1.0734	-1.52832

1	-5.98645	-1.51901	-0.88661
1	-6.88164	2.3396	0.79929
1	-7.55904	0.02531	0.23518
1	-5.17253	4.0959	0.77682
1	0.38992	5.15441	-0.99658
1	-1.2057	6.68366	0.16036
1	-3.46774	5.90165	0.76367
7	-3.51811	-0.98945	-1.63486
1	-3.76515	-1.9661	-1.56041
1	-2.51962	-0.84438	-1.55345

Table S21 Cartesian coordinates for PMMA-Pe S_1□S_0 studied structures using B3LYP/6-31G (d, p) methodology.

	X	Y	Z
6	-1.25258	4.54257	-2.27917
1	-1.41426	4.17954	-3.29879
1	-2.11822	5.13908	-1.98072
1	-0.37231	5.19356	-2.28974
6	-1.03524	3.33619	-1.32504
6	-2.36923	2.52876	-1.31873
1	-3.147	3.19433	-0.93287
1	-2.61356	2.32587	-2.36666
6	-2.51012	1.16788	-0.55128
6	-4.0168	0.78127	-0.74986
1	-4.61077	1.65053	-0.45204
1	-4.1591	0.64989	-1.82681
6	-4.66068	-0.47175	-0.06239
6	-6.11542	-0.52217	-0.64435
1	-6.02508	-0.50001	-1.73434
1	-6.62818	0.39677	-0.34484
6	-7.05206	-1.71298	-0.30908
1	-8.01186	-1.425	-0.76367
6	0.17251	2.52598	-1.82189
1	0.4525	1.73561	-1.1239
1	-0.05758	2.07297	-2.78989
1	1.04526	3.17294	-1.93936
6	-3.89108	-1.7682	-0.35822
1	-3.82637	-1.93082	-1.43626
1	-2.88339	-1.73303	0.05891
1	-4.38308	-2.62926	0.09938
6	-0.70578	3.93167	0.04895
6	-4.72201	-0.27457	1.45896
6	-1.65975	0.12747	-1.29933
6	-6.66501	-2.95179	-1.1167
6	-2.1261	1.30557	0.93004
1	-2.76161	2.05851	1.40585
1	-2.24079	0.36467	1.46878

1	-1.08485	1.60989	1.04684
6	-7.31383	-1.96792	1.18085
1	-6.42825	-2.33477	1.70217
1	-7.63593	-1.03943	1.66285
1	-8.10466	-2.71006	1.31188
8	-1.80526	-0.1368	-2.47584
8	-6.39863	-2.93199	-2.30051
8	0.37088	3.86964	0.61086
8	-4.24405	-1.01962	2.28938
8	-1.75231	4.60959	0.57335
8	-0.72469	-0.46109	-0.52464
8	-5.39323	0.85353	1.79975
8	-6.68928	-4.08899	-0.38737
6	0.11544	-1.42612	-1.18627
1	0.59878	-0.97672	-2.05603
1	0.85649	-1.72228	-0.44546
1	-0.47798	-2.28367	-1.51261
6	-6.37197	-5.2895	-1.11295
1	-5.36378	-5.22989	-1.52946
1	-6.44028	-6.09855	-0.38619
1	-7.08108	-5.44339	-1.92973
6	-5.49616	1.10508	3.21086
1	-6.01979	0.28666	3.71056
1	-4.50386	1.21003	3.65609
1	-6.05884	2.03389	3.30405
6	-1.50684	5.24652	1.83851
1	-0.68563	5.96219	1.75637
1	-2.43531	5.75564	2.09609
1	-1.25188	4.5045	2.59907
6	4.43368	-0.9491	1.06315
6	5.49715	-1.96004	0.89871
6	7.70476	-2.60942	-0.01369
6	6.67256	-1.62551	0.14955
6	8.86967	-2.28661	-0.75491
6	9.01159	-1.03845	-1.31477
1	9.64308	-3.04078	-0.87088
6	8.00356	-0.07071	-1.15661
1	9.90298	-0.78976	-1.88317
1	8.15637	0.89945	-1.6143
6	5.77892	0.68337	-0.26884
6	4.60239	0.34735	0.47687
6	5.87936	1.96175	-0.80885
6	3.57069	1.33098	0.6399
6	4.86382	2.92167	-0.64671
1	6.75859	2.24524	-1.37546
6	3.72511	2.61838	0.06341
1	4.98853	3.9057	-1.08934
1	2.92678	3.34299	0.1919

6	6.83943	-0.32951	-0.43962
6	7.54777	-3.89289	0.56831
1	8.33698	-4.628	0.43726
6	6.41409	-4.19524	1.28583
1	6.29303	-5.17743	1.73323
6	5.3995	-3.23498	1.44769
1	4.52386	-3.51511	2.02121
6	3.26484	-1.21002	1.77305
1	3.11105	-2.18069	2.23005
6	2.25636	-0.24136	1.93074
1	1.36236	-0.49085	2.49456
6	2.40158	1.01023	1.37651
1	1.63637	1.77178	1.48972

Table S22 Cartesian coordinates for PMMA-Dh S_1□S_0 studied structures using B3LYP/6-31G (d, p) methodology.

	X	Y	Z
6	4.65732	-1.93549	-1.36524
1	4.47485	-2.45691	-2.31129
1	4.51014	-0.86404	-1.53048
1	5.7041	-2.10133	-1.086
6	3.70095	-2.48697	-0.27305
6	2.25454	-2.10903	-0.71182
1	2.1996	-1.01629	-0.73696
1	2.13893	-2.4546	-1.74509
6	0.99645	-2.61522	0.07788
6	-0.20223	-1.88228	-0.62251
1	0.05026	-0.81747	-0.65003
1	-0.21115	-2.23162	-1.66028
6	-1.67885	-1.99157	-0.10747
6	-2.51319	-1.19067	-1.16587
1	-2.25116	-1.59083	-2.15041
1	-2.17526	-0.14942	-1.14484
6	-4.06388	-1.18204	-1.09778
1	-4.35445	-0.46592	-1.88201
6	3.92648	-4.00278	-0.1449
1	3.35841	-4.43225	0.68362
1	3.62842	-4.5029	-1.07175
1	4.9823	-4.21615	0.04476
6	-2.15669	-3.44867	-0.00339
1	-2.05521	-3.94831	-0.97059
1	-1.58426	-3.99932	0.74598
1	-3.20283	-3.49889	0.30981
6	4.11193	-1.81845	1.04544
6	-1.82027	-1.33202	1.27207
6	0.85072	-4.11893	-0.20903
6	-4.63813	-2.49962	-1.62065

6	1.09241	-2.29542	1.57797
1	1.18679	-1.21344	1.71779
1	0.21273	-2.63926	2.12448
1	1.958	-2.77655	2.03893
6	-4.67884	-0.68779	0.21851
1	-4.5117	-1.38202	1.04471
1	-4.23959	0.27863	0.49172
1	-5.75888	-0.55241	0.11372
8	0.77984	-4.58725	-1.32717
8	-4.2474	-3.05715	-2.62511
8	4.64494	-2.37816	1.97921
8	-2.24714	-1.87274	2.27056
8	3.86065	-0.48231	1.05041
8	0.80969	-4.88141	0.90574
8	-1.41087	-0.03662	1.27231
8	-5.66166	-2.96491	-0.87158
6	0.6732	-6.29549	0.68104
1	1.52003	-6.67297	0.10227
1	0.65232	-6.74645	1.67335
1	-0.25202	-6.50786	0.13944
6	-6.26612	-4.18072	-1.34454
1	-5.53061	-4.9889	-1.36857
1	-7.06254	-4.40588	-0.63469
1	-6.67201	-4.04107	-2.34983
6	-1.49426	0.63369	2.5442
1	-2.53046	0.66944	2.88976
1	-0.88966	0.111	3.28953
1	-1.10986	1.63872	2.37053
6	4.24764	0.20986	2.25247
1	5.32116	0.10434	2.42634
1	3.9855	1.25468	2.08603
1	3.70691	-0.19321	3.11259
6	-5.87588	3.93577	0.42037
6	-4.49758	3.81376	0.2814
6	-3.67067	4.9542	0.21386
6	-4.29099	6.21706	0.28736
1	-6.49088	3.0409	0.46994
1	-4.05754	2.82263	0.21945
1	-3.6732	7.11077	0.23533
6	-2.21832	4.88941	0.07274
1	-1.72128	5.85744	-0.00038
6	-1.43622	3.78216	0.02715
1	-1.88535	2.79191	0.09963
6	-0.00513	3.82122	-0.1183
1	0.44603	4.80982	-0.20499
6	0.78367	2.71354	-0.14851
1	0.28937	1.74964	-0.02748
6	2.22375	2.65641	-0.31763

1	2.65577	1.66388	-0.20322
6	3.04596	3.69376	-0.61127
1	2.60411	4.68097	-0.74438
6	4.49626	3.65553	-0.79086
6	5.17151	4.85613	-1.08707
6	5.26692	2.47794	-0.68768
6	6.552	4.88626	-1.27102
1	4.59716	5.77588	-1.17294
6	6.64535	2.50921	-0.87191
1	4.78594	1.53144	-0.46078
6	7.29771	3.71159	-1.16422
1	7.04518	5.82773	-1.49835
1	7.21724	1.58851	-0.78873
1	8.37456	3.72971	-1.30763
6	-6.47246	5.19868	0.4946
1	-7.54972	5.28974	0.6026
6	-5.67146	6.33962	0.42646
1	-6.1221	7.32712	0.48125

Table S23 Cartesian coordinates for PMMA-Dm S_1□S_0 studied structures using B3LYP/6-31G (d, p) methodology.

	X	Y	Z
6	-0.39092	-4.6934	1.93317
1	0.24929	-5.44787	1.46525
1	-0.01899	-4.49917	2.94218
1	-1.40046	-5.11015	2.01313
6	-0.39384	-3.40091	1.07239
6	1.06113	-2.84348	1.09824
1	1.28623	-2.58749	2.13703
1	1.72129	-3.67239	0.82185
6	1.48563	-1.61434	0.22058
6	2.96263	-1.32921	0.66838
1	2.95368	-1.25936	1.75989
1	3.53984	-2.22221	0.40878
6	3.77859	-0.09913	0.14137
6	5.21532	-0.31302	0.72914
1	5.53459	-1.31938	0.44217
1	5.13569	-0.29873	1.82007
6	6.37361	0.64106	0.33198
1	7.21091	0.31198	0.96574
6	-0.89254	-3.74988	-0.33839
1	-1.01411	-2.86057	-0.95981
1	-0.18278	-4.42261	-0.82791
1	-1.86522	-4.24562	-0.28699
6	3.80741	-0.02073	-1.39248
1	4.22337	-0.94087	-1.80982
1	2.8071	0.13717	-1.79824

1	4.41248	0.82345	-1.73069
6	-1.39583	-2.44611	1.73943
6	3.18427	1.20827	0.69233
6	1.53207	-2.09232	-1.23944
6	6.88248	0.32272	-1.07333
6	0.55962	-0.40775	0.43273
1	0.57087	-0.10145	1.48173
1	0.86336	0.44639	-0.17309
1	-0.47214	-0.63639	0.16052
6	6.14744	2.13169	0.61533
1	5.37683	2.56375	-0.025
1	5.84052	2.26598	1.65703
1	7.06743	2.69885	0.45524
8	2.18862	-3.04078	-1.6206
8	7.10106	-0.80054	-1.47789
8	-2.47879	-2.13761	1.27628
8	2.79537	2.14117	0.0185
8	-0.9628	-2.03783	2.94596
8	0.76454	-1.35831	-2.07774
8	3.15267	1.2149	2.04234
8	7.10994	1.42761	-1.819
6	0.7913	-1.77223	-3.45373
1	0.43063	-2.79888	-3.55279
1	0.13377	-1.0809	-3.98035
1	1.80755	-1.71615	-3.85083
6	7.62767	1.18052	-3.13679
1	6.92661	0.5759	-3.71704
1	7.75518	2.16268	-3.59154
1	8.5834	0.65369	-3.08234
6	2.59675	2.39629	2.65719
1	3.07518	3.29218	2.25548
1	1.51878	2.44444	2.48958
1	2.80698	2.29251	3.7219
6	-1.81575	-1.13475	3.68125
1	-1.47384	-1.19037	4.71504
1	-1.69676	-0.12016	3.2965
1	-2.85836	-1.44689	3.60089
6	-5.43525	1.93245	-1.53097
6	-4.63487	3.01132	-1.21312
6	-3.61289	2.92473	-0.24648
6	-3.45277	1.6685	0.37463
6	-4.23131	0.56202	0.06743
6	-5.25668	0.66692	-0.89739
1	-6.21844	2.07047	-2.26459
1	-4.809	3.95515	-1.71978
6	-2.73335	4.00108	0.1407
1	-3.98741	-0.36655	0.56685
6	-1.62717	2.48296	1.73423

6	-1.78442	3.76818	1.09232
1	-1.0971	4.54078	1.41543
6	-2.86396	5.35561	-0.5026
1	-2.71736	5.29048	-1.58653
1	-3.86264	5.77527	-0.33703
1	-2.12787	6.05416	-0.10006
8	-0.81791	2.19604	2.60033
8	-2.48925	1.47569	1.32798
7	-6.05668	-0.40891	-1.21321
6	-7.02602	-0.35712	-2.30564
1	-6.61762	0.23964	-3.12699
1	-7.12713	-1.37386	-2.70024
6	-5.99147	-1.66973	-0.47038
1	-5.76504	-1.45814	0.5782
1	-6.99868	-2.1018	-0.48124
6	-4.987	-2.67892	-1.03667
1	-5.19076	-2.88347	-2.0932
1	-3.96223	-2.31624	-0.9373
1	-5.05796	-3.62412	-0.48835
6	-8.40482	0.17159	-1.89035
1	-8.83969	-0.44966	-1.10102
1	-8.33972	1.19535	-1.5113
1	-9.09145	0.16355	-2.74366

Methodology: ωB97XD/def2tzvp

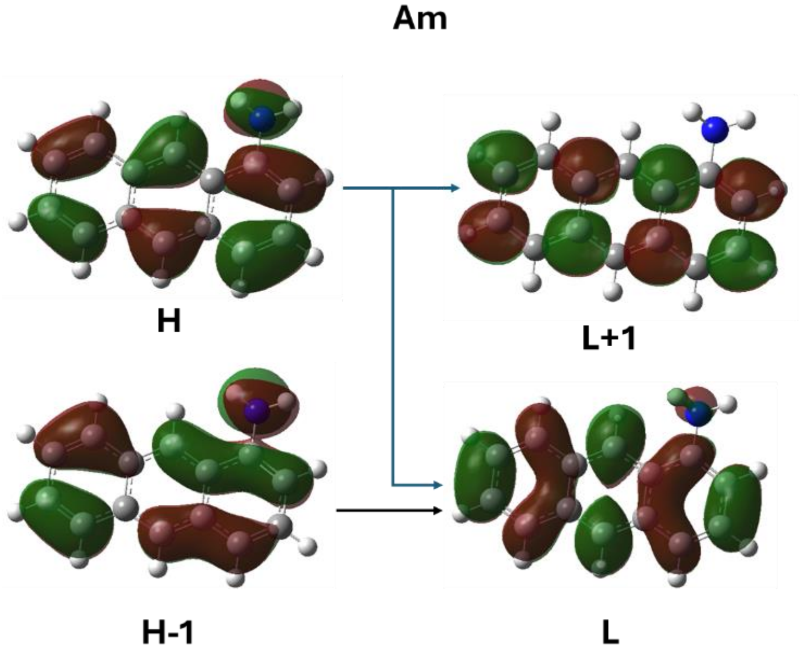

Figure S1. Frontier molecular orbitals of the dye Am at main excitations using the ωB97XD/def2tzvp methodology.

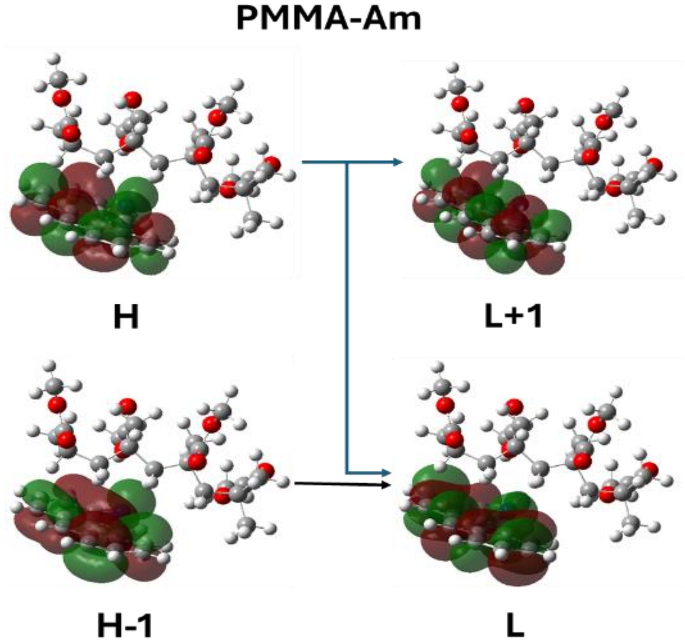

Figure S2. Frontier molecular orbitals of the system PMMA-Am at main excitations using the wb97xd/def2tzvp methodology.

Table S24. Calculated Values of Main Absorption Peaks, λ (nm), Energy Differences, ΔE (eV), Oscillator Strengths, f, Coefficient and Corresponding Main Excitations for the dye Am and PMMA-Am in $CHCl_3$ Solution at ωB97XD/def2tzvp. Distance Between PMMA and Am (Å) is included

λ	ΔE	f	state	C_0	excit.	distance PMMA-Am
			**Am**			
361.9	3.426	0.096	S1	0.6975	H→L	
306.1	4.051	0.012	S2	0.5302	H→L+1	
264.9	4.681	0.016	S3	0.6678	H-1→L	
				**PMMA-Am**		
369.4	3.356	0.119	S1	0.6983	H→L	
307.1	4.038	0.023	S2	0.5459	H→L+1	2.69
268.8	4.612	0.034	S3	0.6673	H-1→L	

Methodology: CAM-B3LYP/6-31G (d, p)

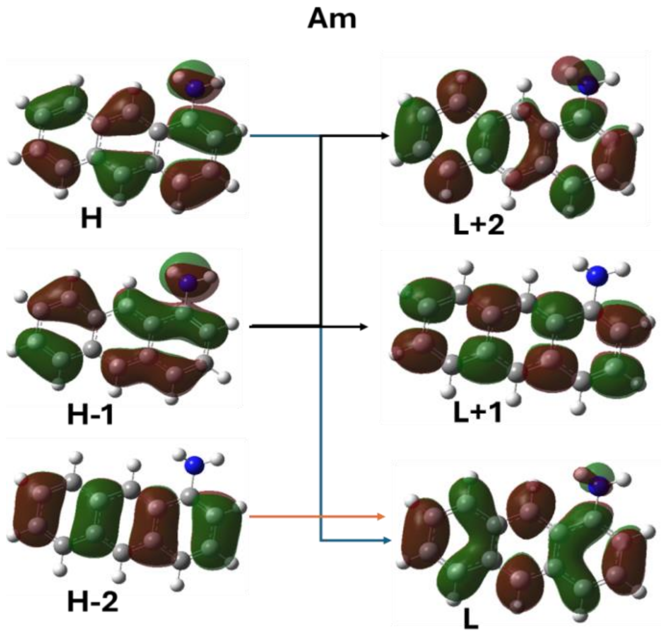

Figure S3. Frontier molecular orbitals of the dye Am at main excitations using the CAM-B3LYP /6-31G (d, p) methodology.

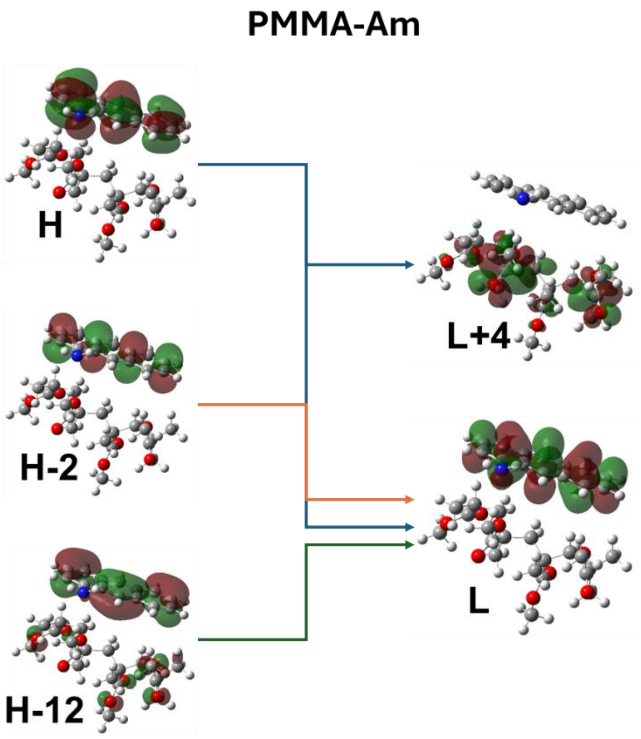

Figure S4. Frontier molecular orbitals of the system PMMA-Am at main excitations using the CAM-B3LYP/6-31G (d, p) methodology.

Table S3. Calculated Values of Main Absorption Peaks, λ (nm), Energy Differences, ΔE (eV), Oscillator Strengths, f, Coefficient and Corresponding Main Excitations for the dye Am and PMMA-Am in $CHCl_3$ Solution at CAM-B3LYP/6-31G (d, p). Distance Between PMMA and Am (Å) is included.

λ	ΔE	f	state	C_0	excit.	distance PMMA-Am
			**Am**			
353.2	3.510	0.104	S3	0.6992	H→L	
232.2	5.339	1.174	S12	0.4658	H-2→L	
211.7	5.857	0.651	S16	0.5092	H-1→L+1	
169.7	7.304	0.137	S38	0.4222	H-1→L+2	
				**PMMA-Am**		
358.6	3.457	0.121	S3	0.7001	H→L	2.54
241.4	5.135	1.557	S12	0.5171	H-2→L	
215.3	5.760	0.334	S20	0.1529	H-12→L	
196.5	6.310	0.206	S33	0.1087	H→L+4	
